# Bioactive Molecules Niosomes Containing a Span 80 and Tween 20 Surfactant Mixture: Obtaining, Physico-Chemical Characterisation and Stability

**DOI:** 10.3390/ph19081241

**Published:** 2026-08-06

**Authors:** Vlad-Andrei Dinu, Romică Crețu

**Affiliations:** Department of Chemistry, Physics and Environment, Faculty of Sciences and Environment, “Dunărea de Jos” University of Galati, 111 Domneasca Street, 800201 Galati, Romania; andreivladd816@gmail.com

**Keywords:** niosomal vesicle, encapsulation, ascorbic acid, fungal dye, antioxidant

## Abstract

**Background**: Niosomes are spherical, non-ionic surfactant-based vesicular carriers structured as lipid bilayers that are widely utilised in pharmaceutical and cosmetic research for integration into topical formulations like gels and creams. **Objective**: This study presents a novel contribution concerning the preparation of niosomes using a mixture of distinct Span and Tween non-ionic surfactants and diverse biomolecules. **Methods**: In this context, novel formulations of niosomes have been prepared by including into their composition a mixture of Span 80 and Tween 20 (in order that HLB = 8.42) non-ionic surfactants with cholesterol (Chol) in a Span 80:Tween 20:Chol = 2:1:1 molar ratio, which is meant to optimise their rigidity and stability, respectively. Their preparation was achieved through three methods: probe sonication, bath sonication and thin-film hydration (TFH). Additionally, the samples were analysed by means of Fourier-Transform Infrared Spectrometry (FT-IR). On the other hand, the antioxidant capacity profile of the formulas of interest was assessed by the DPPH (2,2-diphenyl-1-picrylhydrazyl) radical reduction method. The investigated bioactive molecules were ascorbic acid (AA) and a yellow dye of fungal origin, previously extracted from an *Epicoccum nigrum* strain. **Results**: The average particle size of the niosomes ranged from 2.771 ± 0.808 to 3.397 ± 0.917 μm. Furthermore, high entrapment efficiency was observed for both AA (92.71 ± 0.01%) and the fungal dye (83.54 ± 0.08%), particularly in the formulations prepared through an optimised procedure. The gathered results illustrated that the highest DPPH radical scavenging percentage (48.71 ± 0.05%) occurred when the bath sonication technique was applied. **Conclusions**: These findings demonstrate that the entrapment of active biomolecules depends on the preparation methods utilised. Furthermore, employing fungal dyes represents a promising strategy to simultaneously enhance both the antioxidant and dyeing capacities exhibited by the formulated niosomal colloidal systems.

## 1. Introduction

Ascorbic acid (AA), alternatively known as vitamin C, IUPAC name (5R)-5((S)-1,2-dihydroxyethyl)-3,4-dihydroxy-furan-2(5H)-one, represents a hydrosoluble compound, having four hydroxy -OH groups, with its therapeutic potential being well-described in the literature [1]. This is absorbed, analogously to the monosaccharides, inside the digestive tube, in most cases duodenally and to a minor extent at the stomach and buccal cavity level [2]. Furthermore, it is found in foods, alongside its oxidised form called dehydroascorbic acid, both of them being solely available under exogenous intake due to the lack of gluconolactone oxidase amongst humans, primates and Guinea pigs [3]. Its solubility in water, however, renders it sensitive to water exposure, especially during the washing of fruits and vegetables, leading to slight oxidation. Yet, this process is inhibited by the action of the acidic environment, even at elevated temperatures [2].

Regarding its biochemical functions, the conversion of hydroxy OH•, superoxide O_2_•^−^ free radicals into species devoid of toxicity [4], with collagen biosynthesis at osseous sites [5], is important to mention. Other biological purposes exhibited by vitamin C encompass hormone concentration management regarding insulin, adrenocorticotropin ACTH and adrenaline, and finally the compound in the treatment of methæmoglobinæmia [3,5]; proton acceptance is also dealt with [4]. By way of avitaminosis, scurvy occurs and is associated with two forms, specifically child scurvy (Barlow disease), found in artificially fed infants, and adult scurvy alternatively. The hallmark signs representing this illness are as follows: gingivitis accompanied by missing teeth, punctiform petechiæ, anæmia, astænia, joint pains, and bouts of depression succeeded by raised propensity towards infections [2,5].

Conversely, the chemical structure of vitamin C pinpoints an accelerated rate of degradation by the agency of several external factors like light, elevated temperatures, alkaline environment and the presence of oxygen. In this respective situation, such bioactive molecules, among them being that of vitamin C, are, in multiple instances, encapsulated into a diverse colloidal form. Thus, to overcome this challenge, the development of a niosomal system aimed at optimising the stability of this bioactive molecule was considered throughout this study. The non-ionic surfactants, employed with the aim of increasing the efficacy and stability of said biomolecules, represent amphiphilic molecules capable of modifying interfacial properties [6]. In regard to certain tensioactive agents comprising this class, as esters of Span types of sorbitan with fatty acids and polysorbates with Tween varieties do, the term may even be broadened to emulsifying and solubilisation agents [7,8,9]. With the diminishment in surface tension and in critical micellar concentration (cmc) subsequent to mixing aforesaid surfactants (usually the Rubingh model), either micellar systems—of note as being the ones directly mixed by incorporating surfactant mixtures—or vesicular systems can be created, with the niosomal type highlighted here [10,11,12,13].

Niosomes represent vesicular transport spherical colloidal systems consisting of a lipid bilayer and containing non-ionic surfactants. In contrast to liposomal systems (more extensively described in Table S1 (Supplementary Information) [14,15,16,17,18,19,20,21,22,23,24,25,26,27,28,29,30,31,32,33,34,35,36,37,38] along with other vesicles than niosomes) which consist primarily of phospholipids, the case of niosomal formulations presents the frequent inclusion of cholesterol alternated with its derivatives to achieve optimal bilayer stabilisation [12,39].

The breakthroughs that have been realised in the last decade [40,41] show that, in comparison with liposomes, these showcase increased stability, along with accessibility from a financial point of view, making them better protected against both oxidation and hydrolysis processes. Even though they include synthetic non-ionic surfactants in the stead of lecithins, they might be classified to a certain degree as a variety of liposomes [40]. Moreover, they reveal the capability of co-encapsulating hydrophilic substances inside the core, and lipophilic compounds in the membrane, enabling the amphiphilic molecules to navigate across the pair of compartments [41]. In this way, niosomes are widely utilised in both the pharmaceutical and cosmetic fields of research and development, raising the possibility of their incorporation into gels, creams and consequently, lotions for the purpose of augmenting the potency and stability of bioactive molecules.

In addition to the above, it should be remarked that, apart from niosomes, additional transport vesicles possessing a lipid bilayer also occur. Their respective benefits and limitations are compared and summarised in Table S1 (Supplementary Information).

Considering the various niosome formulation techniques described in the literature and schematised in Table 1 and Table S2 (Supplementary Information) [42,43,44,45,46,47,48,49], prominent methods include the nitrogen N_2_ gas bubbling [43], probe [50,51] and bath [52] sonication, as well as classical methods involving rotary evaporation, namely thin-film hydration [53,54,55] and reverse-phase evaporation [44].

More conventional preparation methods, thin-film hydration (TFH) or hand-shaking [54,55] and reverse-phase evaporation (RPE) [44], entailing the rotary evaporation of solvents such as methanol or chloroform, and, more rarely, albeit of lesser toxicity, ethanol [53,59]. These methods regularly culminate in the generation of niosomes of the multilamellar vesicles (MLV) type and large unilamellar vesicles (LUV) respectively [42,44] having dimensions that cover the interval of 100–1000 nm (nanometres) in general, at times reaching 3000 nm, in comparison with those of small unilamellar vesicle (SUV) kind with 10–100 nm [60]. Those specific formulations obtained through these procedures are readily optimisable with the aid of ultrasonication for the purpose of diminishing vesicular dimensions. While TFH presupposes the production of a dry thin film to be hydrated with an active-substance-containing aqueous phase, RPE involves the emulsification of the aqueous phase into the organic one [57], implying the redissolution of the film. As a special variation in thin-film hydration, the transmembrane pH gradient method involves the use of an acidic-pH buffer solution, followed by the addition of an aqueous drug solution during freeze–thaw cycles homogenisation, and finalised with the addition of a buffer solution having a neutral-to-slightly alkaline pH [29].

As for SUV-type niosome generation methods that bypass the necessity of solvating surfactants and cholesterol at one time, both types of ultrasonication [42], whether by probe [50] or in bath [52], usually drive the diminution of niosome dimensions, and can even be applied to the optimisation of niosomal formulations achieved through other means [58]. Given that the ultrasound emitted from the probe is applied directly inside the flask, as opposed to from its vicinity in the case of the bath, the production of even smaller vesicles can thereby be argued.

The stability of the niosomal membrane is optimised by the inclusion of cholesterol [61], meaning that the hydrogen bond formation is mediated with the surfactants utilised [62]. This effect is even more pronounced in ternary systems utilising binary surfactant mixtures [63], something that favours the subsequent organisation of the lipid bilayer. Furthermore, the synergistic action manifested by surfactants and cholesterol [37] determines a significant rise in the entrapment efficiency of bioactive compounds [42].

Similarly, one may substitute cholesterol with a derivative of it, Solulan G_24_, with the goal of attaining a matching stability, whereby polyhedral niosomes are formed during heating [64]. Still in strengthening vesicle stability, an essential role is played by charge inducers [24,65], notably dicetyl phosphate (DCP), stearyl amine and stearyl pyridinium chloride.

Regarding the blending of non-ionic surfactants for niosome formulation, particularly those of the Span and Tween series, a vast number of studies have investigated combinations of structurally identical analogues [66,67]. However, data concerning the employment of non-matching Span and Tween surfactants—such as the combination of Span 60 and Tween 40—remain comparatively limited [68].

With reference to the study of the quenching phenomenon aided by spectrofluorophotometry combined with fluorescence markers, via niosomal probes encasing a cobalt-calcein complex, these showcase a diversity of models representing entrapment efficiency (% EE) [69,70,71].

The utilisation of niosomal systems optimises the delivery of a broad range of therapeutic agents, on account of their comparable stability and elevated entrapment efficiency. An approach like this has demonstrated an inherently superior therapeutic efficacy across an extensive variety of applications, including but not limited to ocular delivery of pilocarpine hydrochloride [58], oral administration of paclitaxel [72], vaginal administration of insulin [73] and subcutaneous transport in the case of minoxidil [54].

The encapsulation of a number of antioxidant molecules, which besides this property are also renowned for their dyeing ability, into niosomes has led to coloured niosomes, namely curcusomes [74,75,76], as well as to improvements in antioxidant capacity [77]. Among the methods involved in gauging the antioxidant activity displayed by molecules encapsulated in niosomes, DPPH [78] and lipid peroxidation LPO [79] assays are worth mentioning. In this instance, the incorporation of antioxidant molecules has driven an enhanced 1,2-diphenyl-2-picrylhydrazyl (DPPH) radical scavenging capacity displayed by a predominantly lipophilic Span 80: Span 85 blend, compared to its isolated counterparts, and has even led to decreased LPO values. These findings confirm the potential of niosomes as antioxidants in combating free radicals.

**Figure 1:**
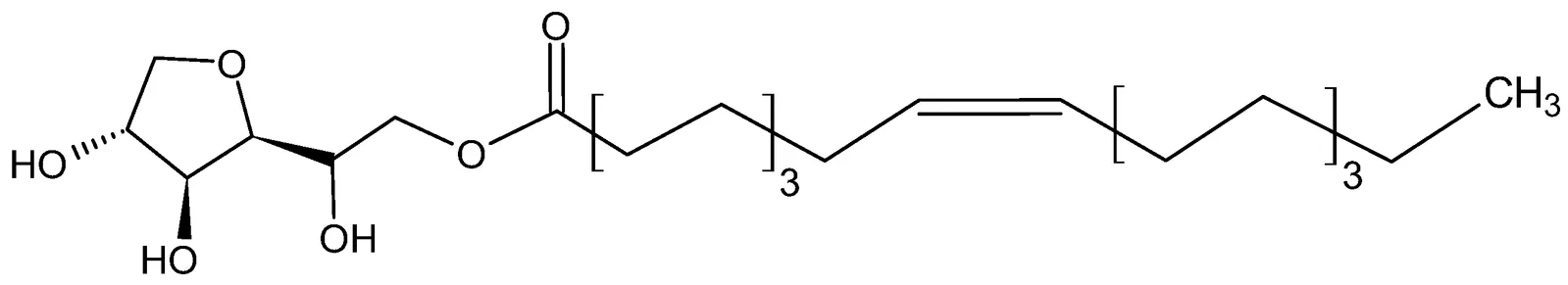
Structural formula of the sorbitan monooleate (Span 80) surfactant (structurally adapted from Sadeghi et al., 2013 [8]).

**Figure 2:**
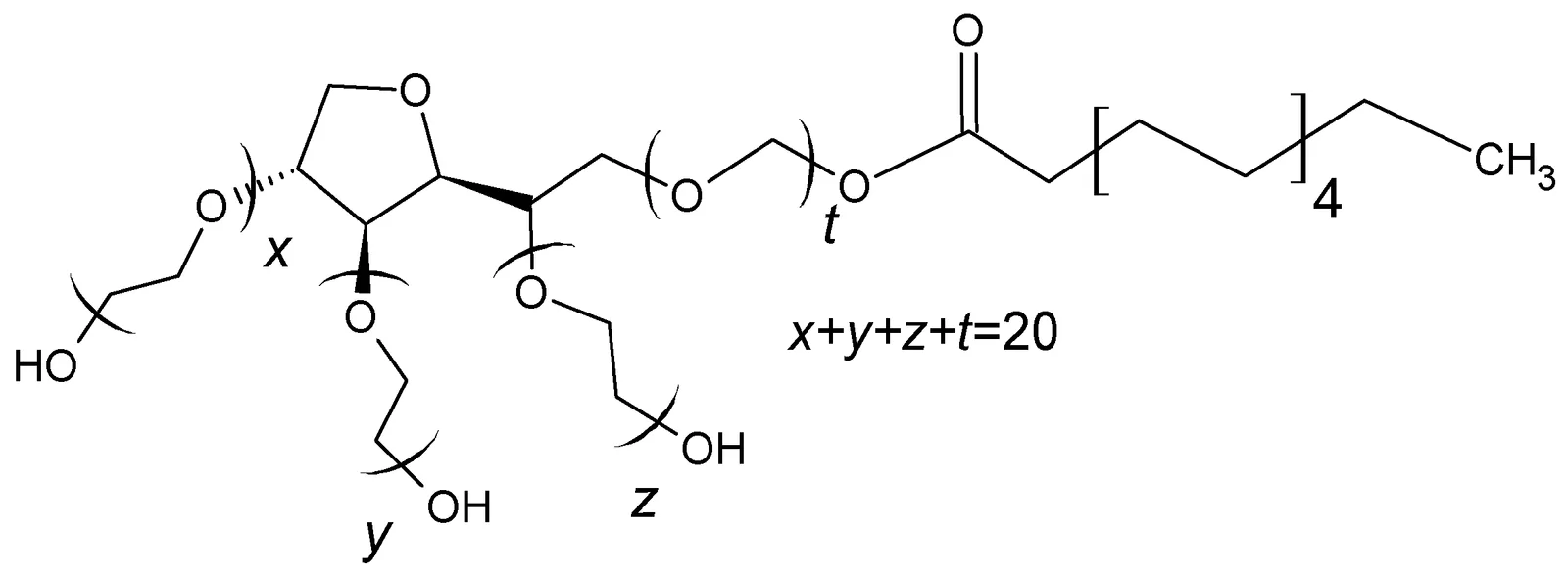
Molecular structure of the non-ionic Tween 20 (polyoxyethylene 20 sorbitan monolaurate) tensioactive (modified from Tan et al., 2016 [9], and adapted from Sadeghi et al., 2013 [8]).

In light of the aforementioned, the main aim of this study is to formulate ascorbic acid (AA) niosomal suspensions by employing a mixture of Span 80 (Figure 1) and Tween 20 (Figure 2) across various preparation techniques, evaluating their potential for applications in the pharmaceutical, cosmetic, and, consequently, cosmeceutical fields [80].

Such a mixture had previously been evaluated with regard to surface tension [10] as part of studies conducted in order to address multiple shortcomings pertaining to AA, including thermolability and photosensitivity, as well as to assess the stability of these formulations over time.

**Figure 3:**
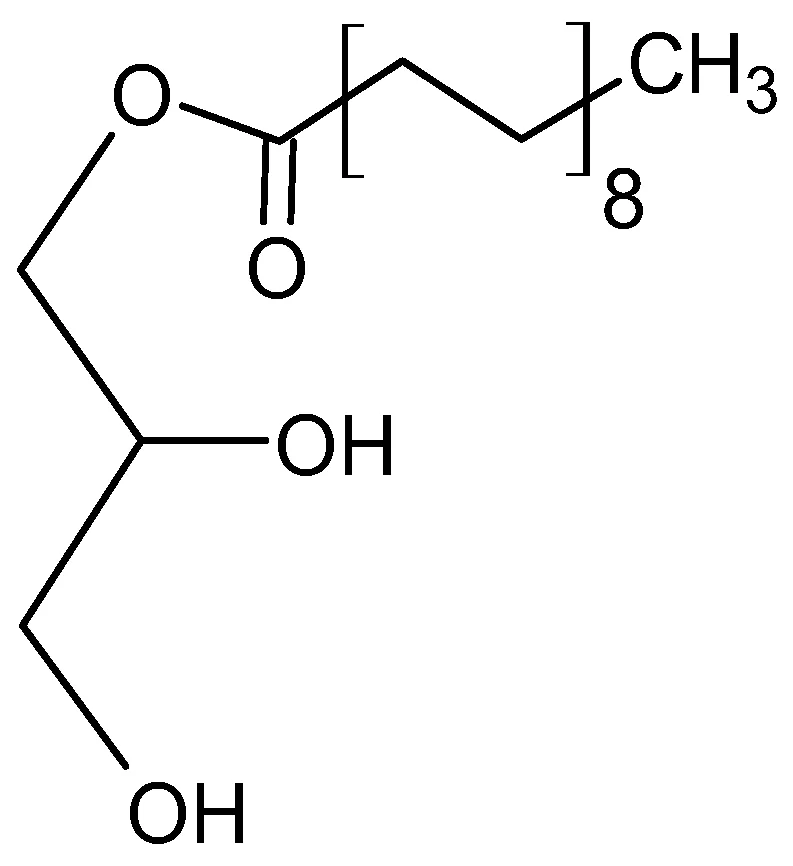
Structure of glyceryl stearate.

Additionally, this research offers significant novelty by addressing a poorly explored area, specifically the encapsulation of a fungal dye. In this context, we have encapsulated a previously extracted fungal dye rich in both carotenoids and flavonoids [81] into such niosomes with the intention of emphasising particularities correlated with substance entrapment, whether hydrophilic or lipophilic. Particular to this study is the involvement of three of the methods described above for the niosomal vesicle preparation at a given surfactant mixing ratio: probe sonication, bath sonication and thin-film hydration (TFH). This is an approach distinct from other conventional studies addressing two preparation methods combined with various non-ionic surfactant mixing ratios. These methods were chosen because of their elevated entrapment efficiencies and their ability to generate high-stability vesicles alike [42,44]. Furthermore, specific to this study, we have even resorted to the use of glyceryl stearate (Figure 3) as an alternative to charge-inducing agents, such as dicetyl phosphate (DCP).

## 2. Results and Discussions

The development of niosomal systems for the efficient encapsulation and controlled release of bioactive molecules is influenced by the hydrophilic-lipophilic balance (HLB) value of the surfactant mixture used. Most studies reported in the literature have focused either on the use of individual non-ionic surfactants [82] or on selected binary formulations in an equimolar mixture [83] or on formulations characterised by various ratios [84] and HLB values at the extremes, namely strongly hydrophilic (HLB > 10) or predominantly lipophilic (HLB ~ 5) [75]. In contrast, the present study adopts a rational formulation approach, based on the identification and evaluation of an optimal HLB (8.42), in order to obtain favourable encapsulation characteristics for the niosomes. The results of the present study underscore the advantages of this specific system, particularly in achieving favourable niosomal encapsulation characteristics, such as entrapment efficiency. This behaviour is attributed to the optimised balance between the rigid hydrocarbon chain of Span 80 and the highly hydrophilic polyoxyethylene chains of Tween 20.

**Figure 4:**
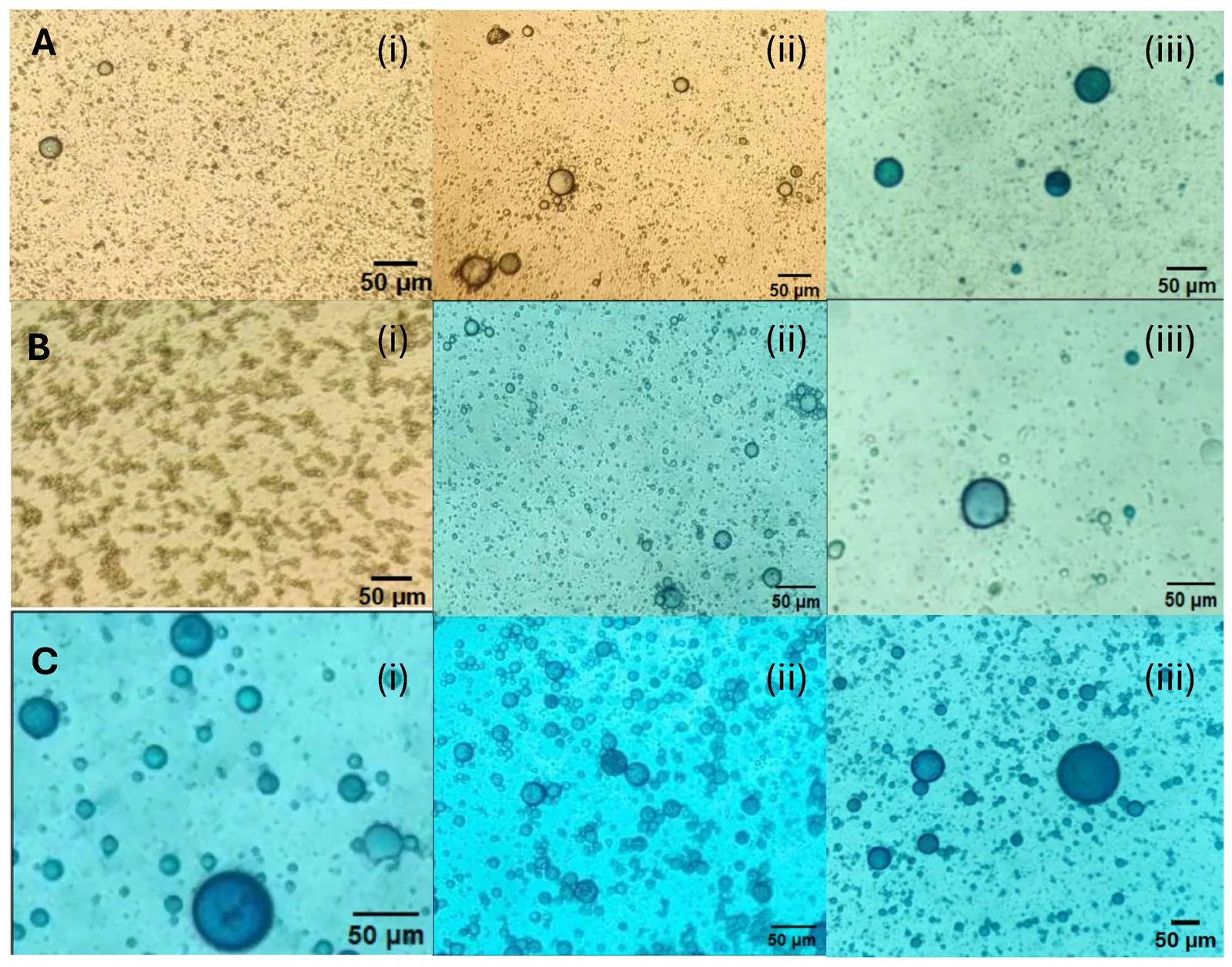

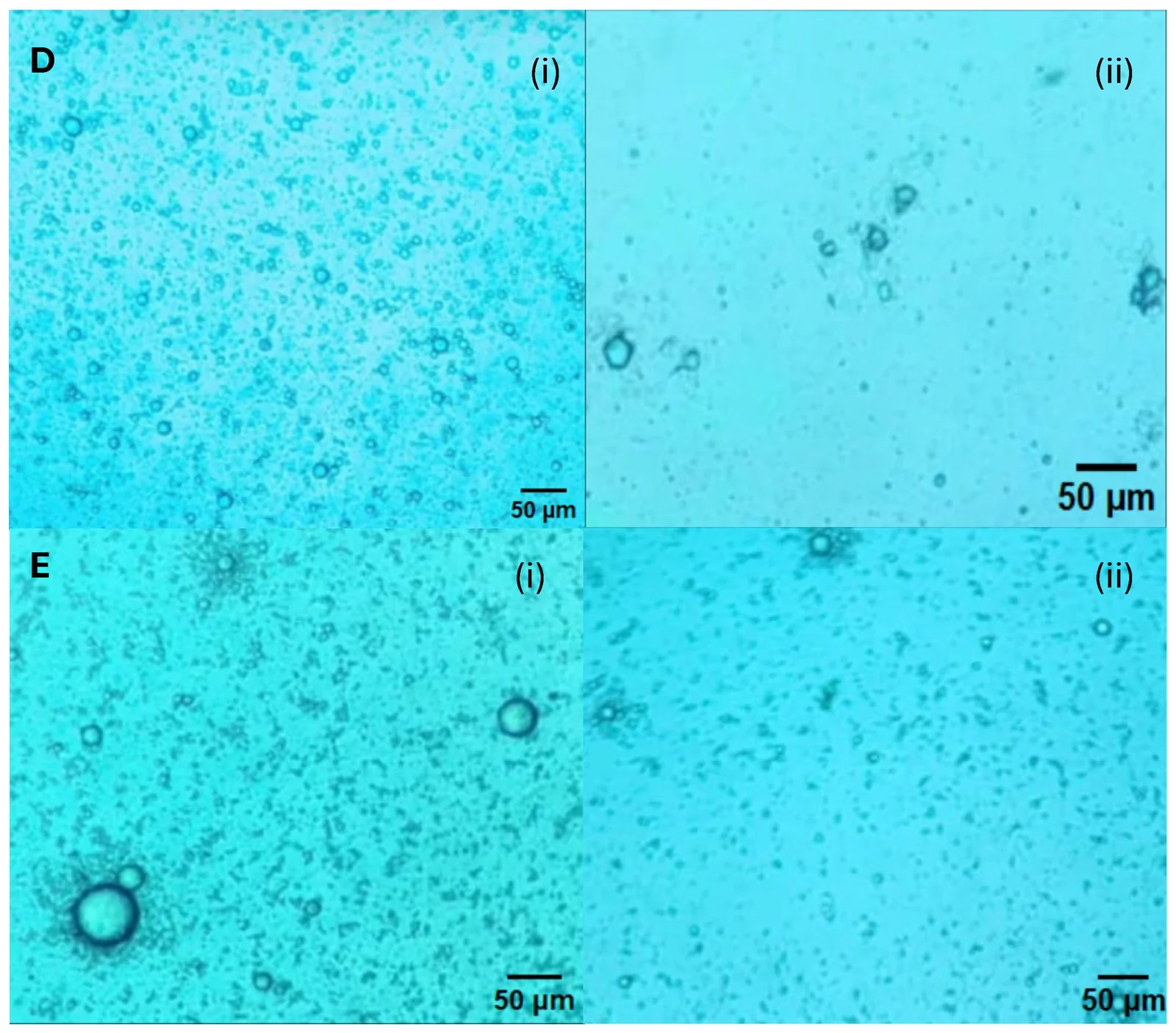
Typical micrographs illustrating the niosomal formulas at diverse time intervals: (**A**)-NB: (**i**) on the first week, (**ii**) the fourth week, (**iii**) the sixth week; (**B**)-NA1: (**i**) on the first week, (**ii**) on the third week, (**iii**) on the fifth week; (**C**)-NA2: (**i**) on the first week, (**ii**) on the second week, (**iii**) on the fourth week; (**D**)-NA3: (**i**) on the first week, (**ii**) on the third week; (**E**)-Ndye: (**i**) on the first week, (**ii**) on the second week; moments of inflexion in the vesicular morphology.

In view of the physico-chemical and morphological characterisation of Span 80–Tween 20 niosomes, be they blank, AA-containing or fungal dye-containing, the Microsoft Excel program was used to register the collected data. The analysis of niosomal formulations, with respect to vesicular morphology, evidences a clearly outlined demarcation boundary at the edge of the particles, as a signature of the lipid bilayer. In this way, we have demonstrated the successful formation of niosomes, as validated, for example, in the case of the very first two niosomal formulations, amongst which a homogeneous vesicular morphology can be observed (Figure 4).

Furthermore, the analysis of all the microphotographs revealed a predominantly spherical morphology for the vesicles present within the niosomal formulations. Regarding the ascorbic acid samples (NAi) prepared with probe sonication and bath sonication, respectively, an initial instability was observed (B-i and C-i). This behaviour correlates with the relatively large dimensions of certain vesicles, demonstrating that these niosomes exhibit increased sizes during the first week post-preparation.

Conversely, while in the instance of the niosomal vitamin C formulation involving bath sonication, particle size variation situates itself within an extremely restricted range over time (C-ii), the sample formulated with the help of the probe indicates a much more pronounced rise in average size over time (B-ii). This finding stresses the fact that the type of formulation technique has an obvious impact on the niosomes’ stability.

**Figure 5:**
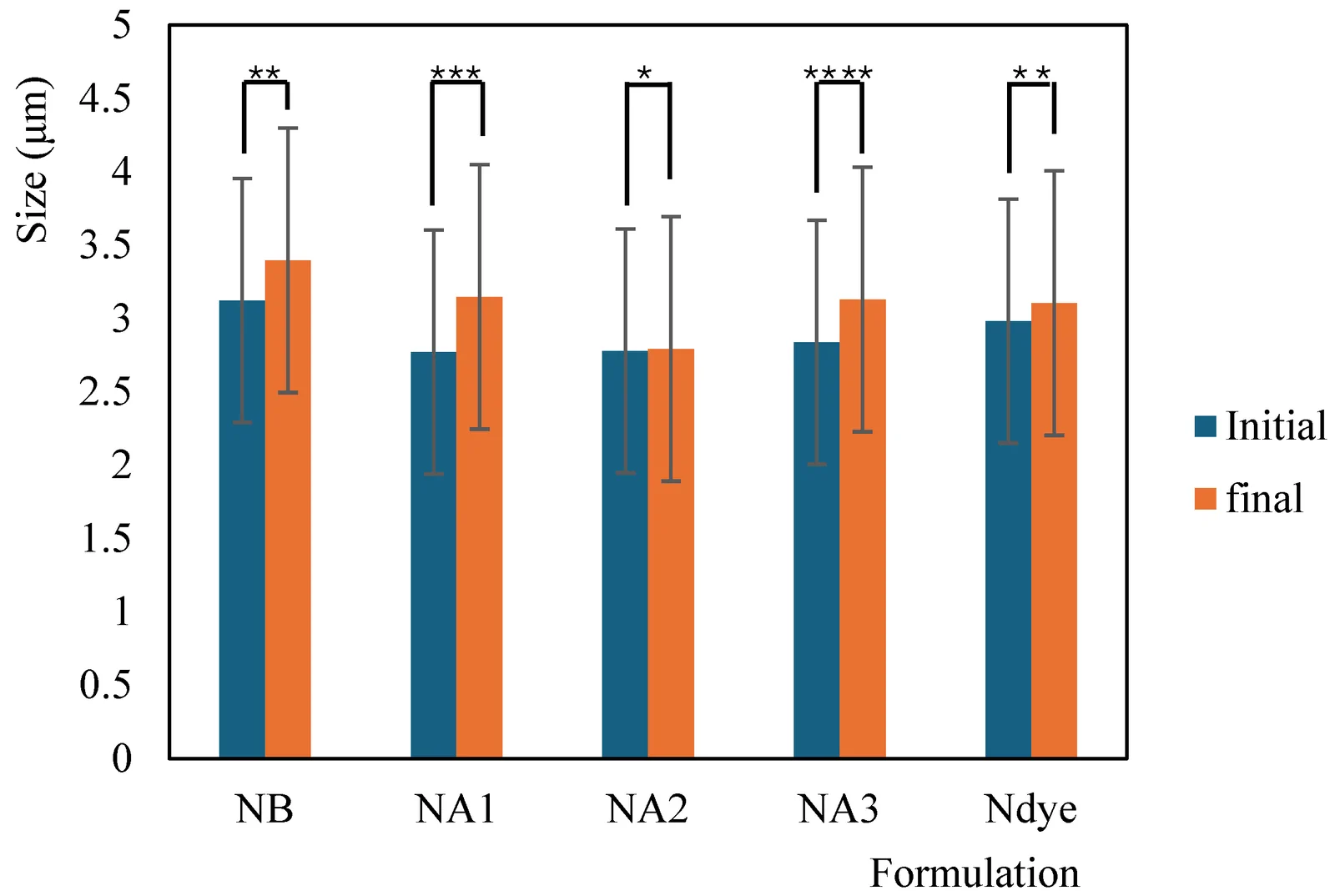
Average size of niosome vesicles. The symbols appear as presented in Figure 4. Values are expressed as mean ± SD of triplicate readings; statistically significant differences: * *p* < 0.05, ** *p* < 0.01, *** *p* < 0.001, and **** *p* < 0.0001.

The average vesicle size ranged from 2.771 ± 0.808 to 3.124 ± 0.892 μm as initially determined after formulation, and from 2.793 ± 0.939 to 3.397 ± 0.214 μm as evaluated at the final time point (Figure 5), as a function of the inflexion point beyond which no significant modifications occurred in their morphology.

The final time point of their determination extended to a maximum of five weeks, an aspect encountered with the ascorbic acid niosomes produced with the probe sonication technique (NA1).

The obtained results show that, in addition to typical niosomes, they can also appear as large unilamellar vesicles that may extend into the micrometric range, with diameters even reaching several micrometres, depending on the preparation method [62]. These niosomes were produced using techniques that initially generate larger vesicular systems, including micron-sized multilamellar niosomes, rather than nanometre-sized ones. Similar observations have been reported in previous studies [85], where the use of such methods and techniques produced micrometre-sized vesicles. Although the larger size of these niosomes may reduce systemic bioavailability compared to nano-scale carriers, it can offer distinct advantages. These specific characteristics—such as a higher loading capacity for bioactive molecules [86], a slower release profile [66] and enhanced local retention for topical or localised applications [87]—support the concept that such systems, when viewed through the lens of lamellarity, are still classified as niosomes.

Furthermore, considering the presented data (Figure 5), a noticeable similarity can be highlighted in the dimensions of the initial niosomal formulations associated with the utilisation of the probe sonication and ultrasonication bath methods, individually (2.771 ± 0.808 μm and 2.778 ± 0.732 μm respectively). Even if their dimensions exceed 1000 nm, they could still be classified as LUV-type niosomes [60,75].

Among other things, it should be noted that there is an increase of 13.57% in the average size of the niosomes resulting from the probe sonication technique, whilst in the case of the ones obtained using the sonication bath, the increase in their average size was markedly smaller (0.54%). These respective increases are, in all probability, a consequence of the inclusion of vitamin C into the niosomal bilayer, and, at the same time, of the resulting change in the structural organisation of the bilayer.

Moreover, this increase in niosomal sizes is attributed to the hydration phenomenon of the bioactive compounds and their molecular interactions within the matrix. Nevertheless, while considering the 0.54% variation in the NA2 case, it can be asked whether the particle size increase following active biomolecule incorporation can be attributed to the ultrasonication technique used in the formulation of niosomes. In this way, a pronounced stability can be remarked with respect to the time evolution of these niosomes, consequently suggesting that the use of bath sonication is the optimal method for achieving formulations like this kind. However, as for the niosomes containing the fungal pigment, an increase of only 0.121 μm is identified after two weeks from their formulation, with their morphology (Figure 4E(ii)) remaining ulteriorly unmodified. Such results exhibit statistical significance (*p* < 0.05), which denotes good stability of the formulated niosomes.

**Figure 6:**
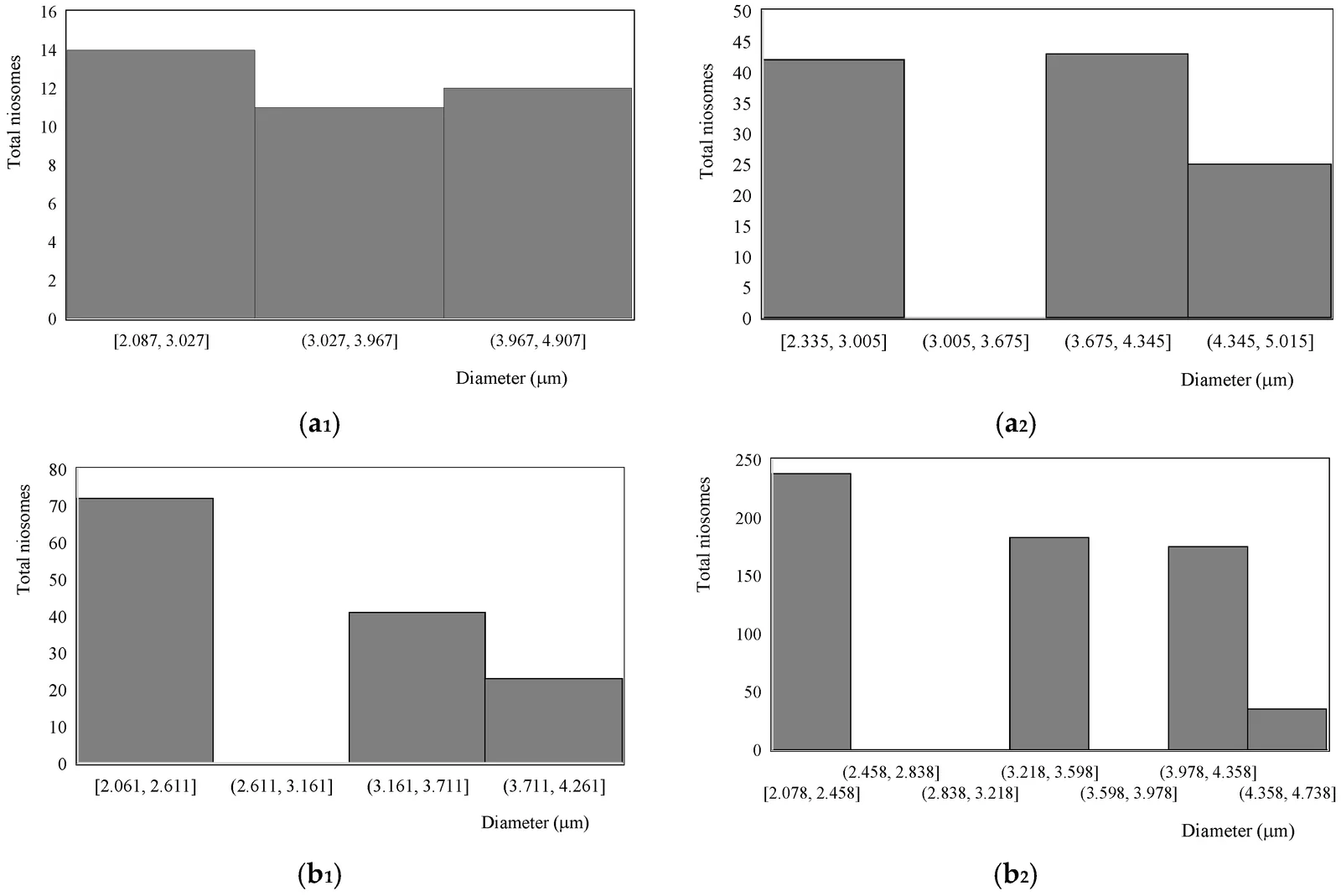

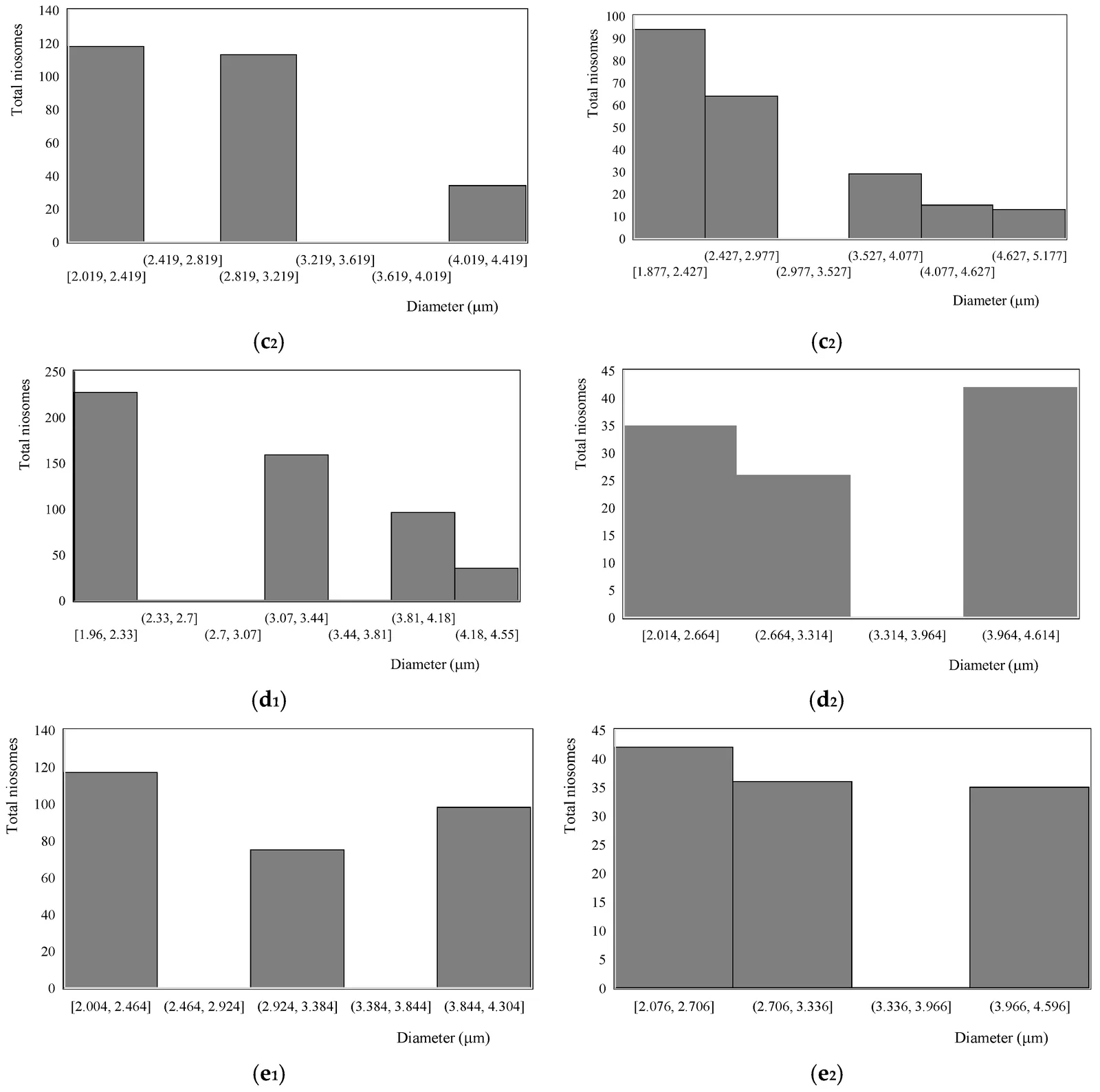
Size distribution of niosomes having bioactive molecules generated via diverse formulation techniques: initial NB (**a_1_**), final NB (**a_2_**); initial NA1 (**b_1_**), final NA1 (**b_2_**); initial NA2 (**c_1_**), final NA2 (**c_2_**); initial NA3 (**d_1_**), final NA3 (**d_2_**); initial Ndye (**e_1_**), final Ndye (**e_2_**). The symbols are: NB-Blank niosomes (without bioactive molecules); NAi—Niosomes containing vitamin C, i = 1—3 depending on the method involved in their preparation—probe sonication (1), bath sonication (2) and thin-film hydration (3); Ndye—Niosomes containing fungal dye.

This is supported by the broad size distribution in Figure 6a–e.

The size distribution of the bioactive molecules niosomes reveals a strong correlation with the microscopic observations and is consistent with the niosomes diameters. It can be ascertained that the niosomes’ diameter first reaches over 4 μm for the values of all of the studied samples and even above 5 μm in the case of the final formulations with NB and NA3. An increase like this in the vesicle size could be linked with an elevation in surface free energy.

Besides that, in the case of the initial niosomal formulation, a not necessarily homogeneous distribution of the vesicle population can be remarked. By contrast, regarding the niosomal formulation with ascorbic acid, obtained using bath sonication (NA2), the size distribution analysis pinpointed a narrow distribution associated with a vesicle population that is relatively homogeneous in dimensions. This fact emphasises the microscopic observations regarding their superior stability.

**Figure 7:**
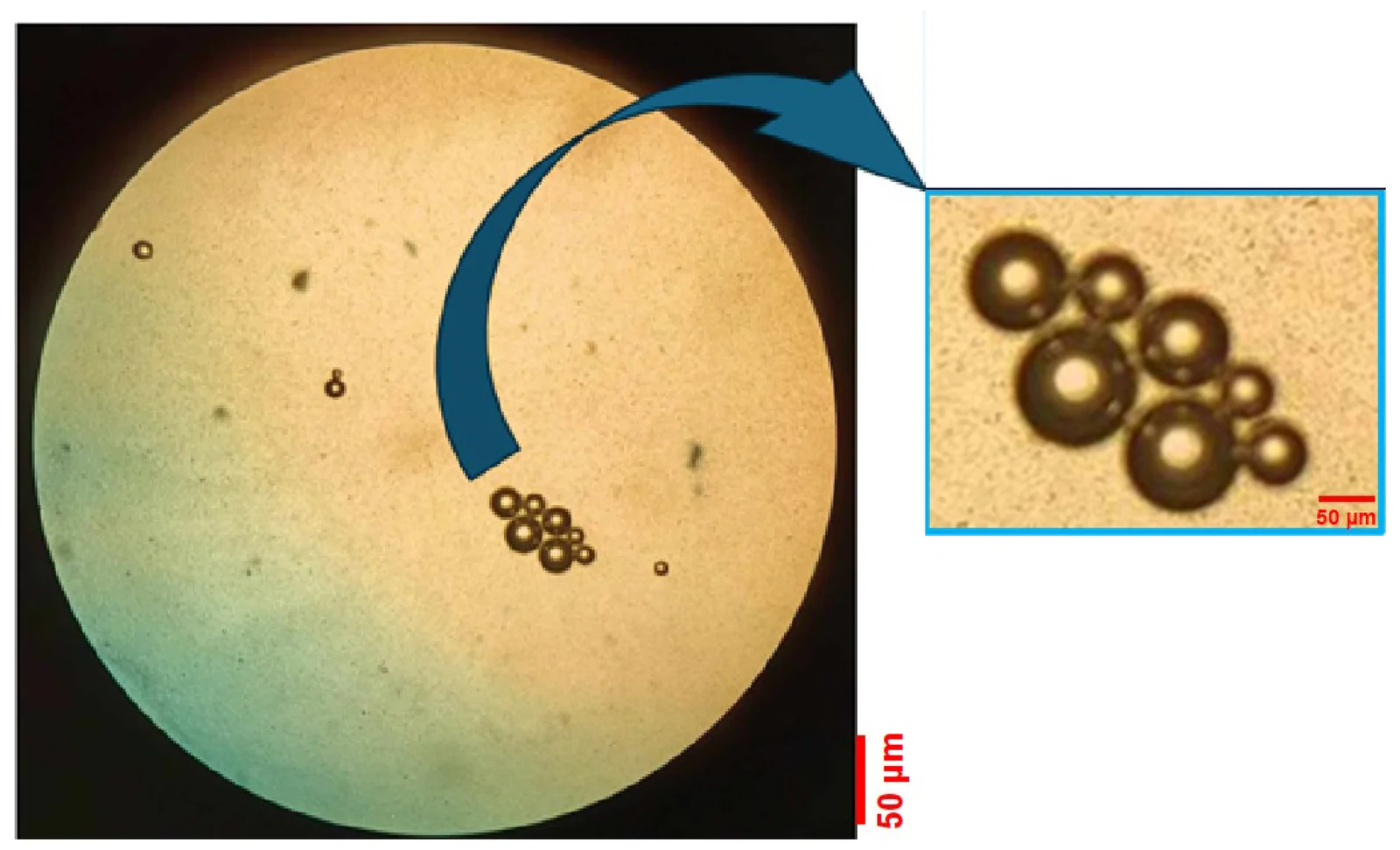
Magnified view of multilamellar (MLV) niosomes found inside the NB formulation (4×), determination following the very first week following preparation.

Similarly, aside from LUV-type vesicles, one would eventually notice MLV-type niosomes, in the way they are highlighted in Figure 7, as exemplified for NB. While ultrasonication did not result in SUV particles, in this particular instance the average dimension of such vesicles was approximately equal to 3.124 ± 0.832 μm. These micron-sized dimensions indicate the formation of large unilamellar (LUV) or multilamellar vesicles (MLV). This outcome aligns with the specific selection of mild sonication parameters designed to preserve this macromolecular structure, which is crucial for maximising the entrapment efficiency of the dye.

**Figure 8:**
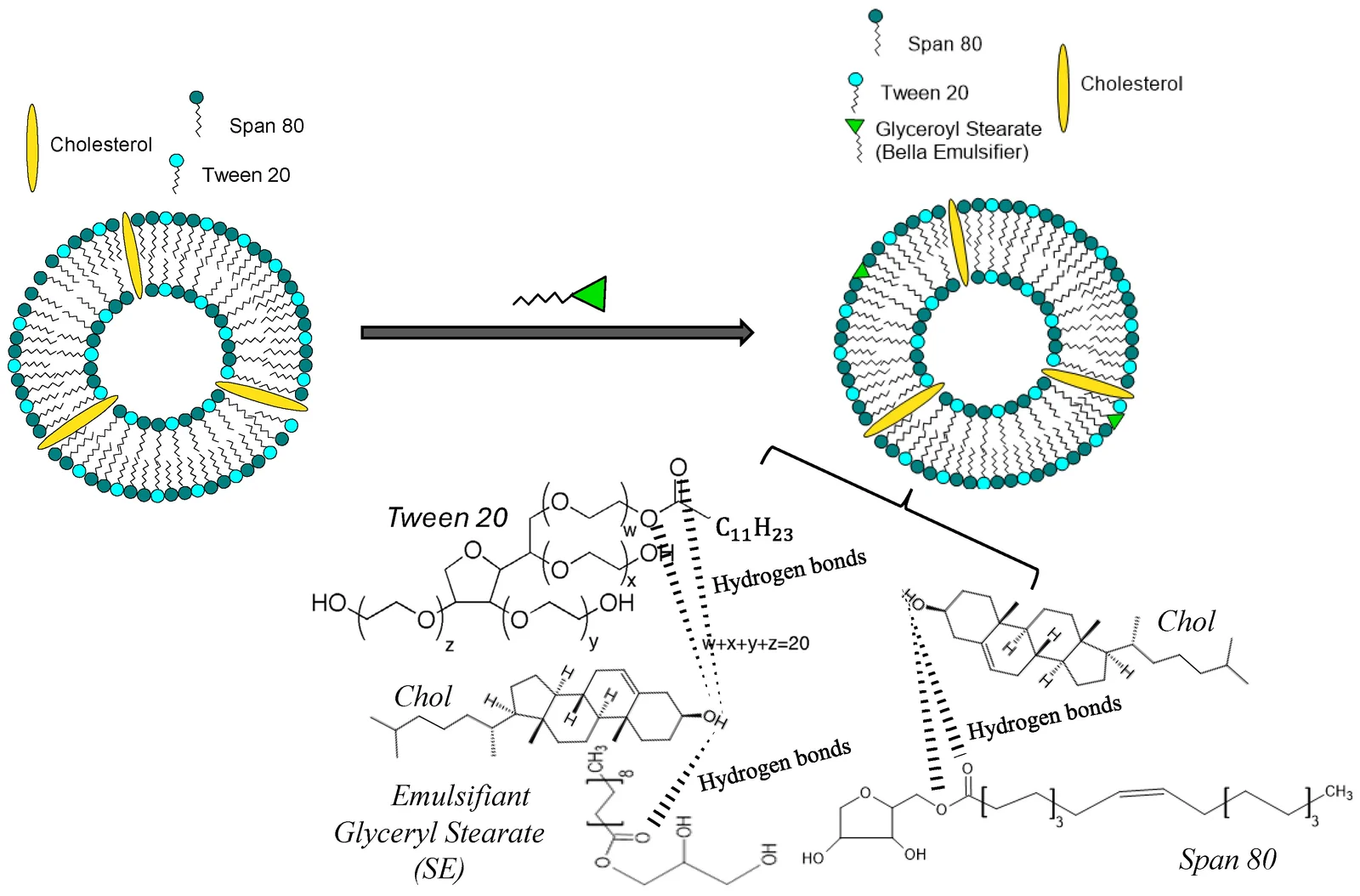
A hypothetical model of the proposed mechanism, highlighting the interactions amongst non-ionic surfactants, glyceryl stearate and cholesterol molecules in the NB formulation.

**Figure 9:**
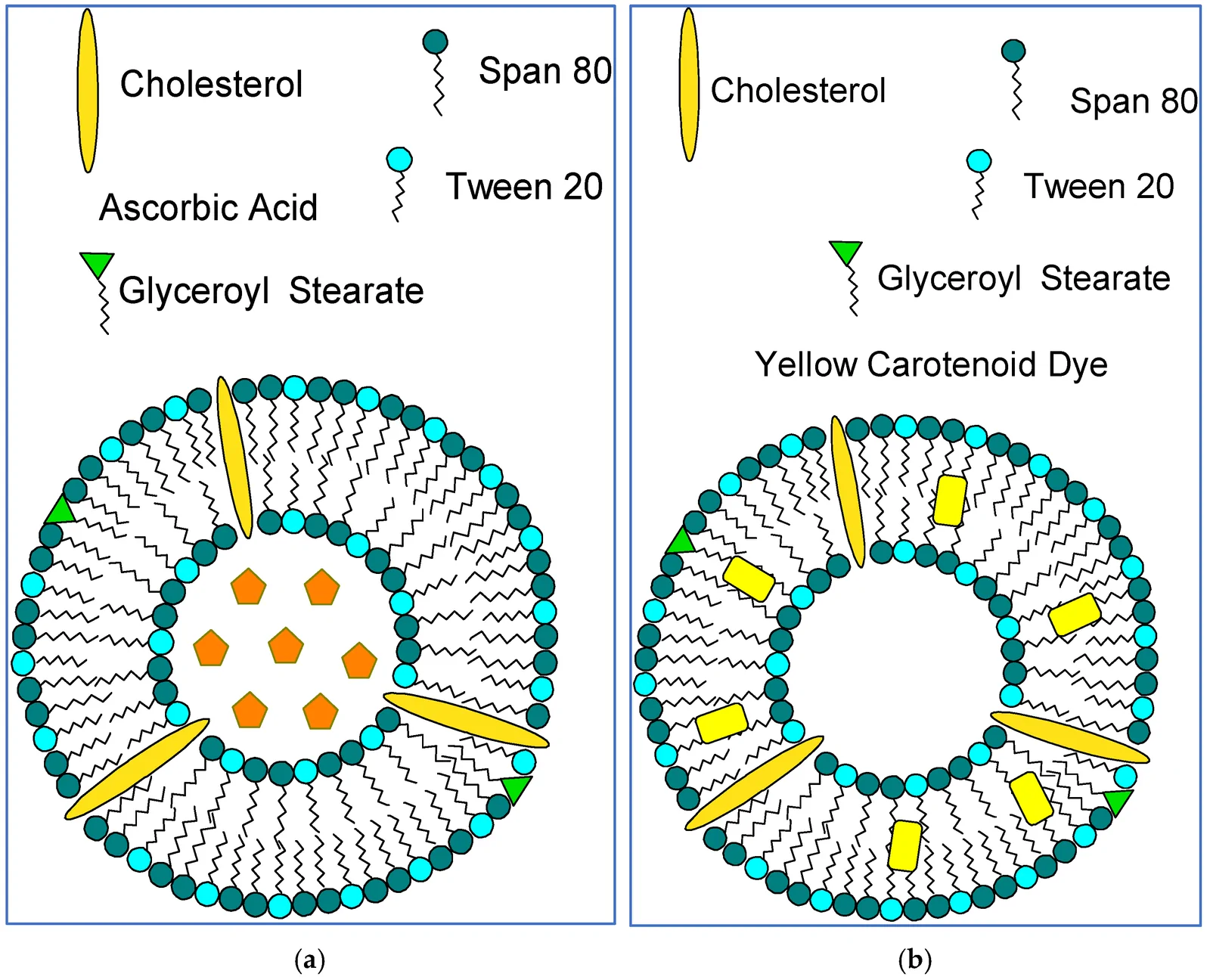
A hypothetical model of niosomes with non-ionic surfactant. Hydrophilic bioactive molecules, such as AA, can be encapsulated in niosomes in the hydrophilic inner part (**a**). Cholesterol and fungal dye could be incorporated into the hydrophobic bilayer region (**b**).

The collected results prove a substantial stabilisation of the bioactive molecules by virtue of the combined system comprising non-ionic surfactants and cholesterol molecules (see the hypothetical models presented in Figure 8 and Figure 9a,b). The incorporation of the specific fungal dye studied can significantly influence membrane packing and rigidity. Given that the encapsulated agent is a bulky, hydrophobic molecule that inserts directly into the lipid bilayer (Figure 9), the larger vesicle volume and membrane surface area are essential to accommodate its structural requirements. Furthermore, our results validate that extremely hydrophilic bioactive molecules, with ascorbic acid taken as an example, could be exclusively immobilised in the hydrophilic compartment of the niosomes (Figure 9a) by way of a variety of techniques. Beyond that, lipophilic bioactive molecules, such as the ones contained in the fungal dye, may be successfully entrapped inside the lipid bilayer (hydrophobic) compartment of the niosomes (Figure 9b). The mechanisms presented are working hypotheses based on the specialised literature. In this context, these results regarding the fact that polysorbate niosomes have good capture capacities for hydrophilic molecules are similar to those highlighted in the specialised literature [62]. In the case of these types of niosomes, the possibility of bioactive molecules forming hydrogen bonds increases. This leads to an increase in niosomal cohesion.

In the pH-metry (Table 2), the determinations were carried out over the course of several weeks; the average values, following a prolonged stretch of time, situated themselves in the pH range of 6.6–6.96 pH units for the ultrasonic formulations, thus proving them to possess a marginally acidic character. As for the niosomes prepared by way of the TFH method (NA3 and Ndye), mean values ranging from 1.902 ± 0.008 to 2.017 ± 0.019 were registered, corroborating in this way a starkly acidic character.

**Figure 10:**
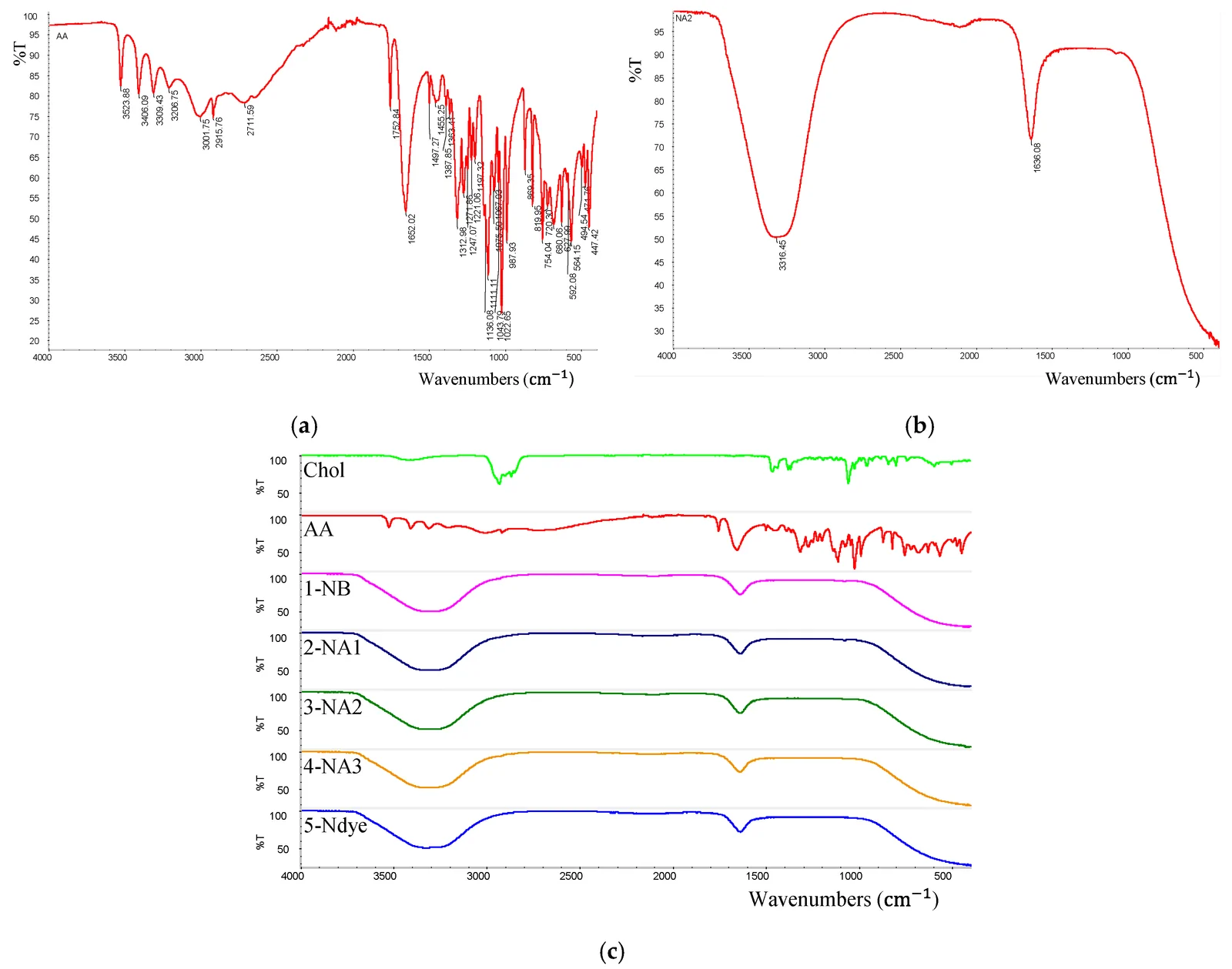
The FT-IR spectra of the bioactive substance and of the encasing niosomes, more precisely AA (**a**) and NA2 (**b**), presented comparatively in terms of the five niosomal formulations and of cholesterol, belonging to the AA by isolation (**c**).

The FT-IR spectra (ATR method) of the bioactive molecules, alongside those of the niosomal formulations, are outlined in Figure 10, by comparison with the spectra exhibited by the hydrophilic and lipophilic substances included in their composition.

In the FT-IR spectra of these niosomal formulations, the characteristic absorption bands assignable to the moieties of the bioactive molecules present were identified in accordance with the literature recommendations [88,89,90] and included in Table 3.

The FT-IR spectra indicate a virtually identical pattern across each of the five niosomal formulations, a similar case being encountered in the literature, namely with Span 60 liponiosomes [38]. This fact corroborates the proper formation of the vesicles, along with the successful encapsulation of the bioactive molecules. At the same time, the absence of new peaks did entail the lack of chemical interactions between the bioactive molecules and the excipients, just as is signalled throughout the scientific literature [92]. In accordance with data from Figure 10, one can identify an absorption band occurring at the wavenumber of 1752 cm^−1^ corresponding to the lactone ring (five-membered ring) specific to ascorbic acid, an attribution that is in concordance with the data reported by Khan et al. [91]. In addition to the abovementioned, the existence of hydrogen bonds could also be noted through the broad bands within the 3000–3600 cm^−1^ range among the five niosomal formulas, something that explains the stability of the membranes comprising a Span/Tween/cholesterol ternary system, an aspect established as being more thermodynamically stable compared to a niosomal bilayer encompassing a binary Span/Tween system [63]. The incorporation of the fungal dye in the niosomal formulation does not induce substantial alterations when compared with the blank formulation. One may, however, notice the occurence of a number of peaks in the spectral region ascribed to the absorption bands stretching across the 1400–1800 cm^−1^ interval which could be characteristic of specific functional groups (C=C vibration band associated with conjugated double bonds) and of the molecular structure of a number of pigments, particularly β-carotene (at approximately 1636 cm^−1^, C=C [91]), present in the fungal colourant composition. Besides these, the visible frequencies representative of the FT-IR spectra peculiar to these niosomes could be viewed in the spectral area of 3300–3400 cm^−1^, most likely as an effect of the presence of xanthophylls, and even of flavonoids, as part of the fungal colourant constitution. Furthermore, in the case of the wavenumber of 1636.09 cm^−1^ it is the C=C vibration band that could be attributed to the existence of the olefinic double bond from the oleic acid remnant of the Span 80 surfactant structure in the case of each of the five niosomal samples, with the cis (Z) conformation of the hydrogen atoms having been proven by a broad band having a transmittance minimum at 900 cm^−1^, whereas the ascorbic acid displays such kind of a bond at 1652 cm^−1^.

Thus, the information obtained through FT-IR analysis suggests or supports the success of the encapsulation process, corroborating the other data provided by various characterisation methods used.

Furthermore, determinations related to the entrapment efficiency (%EE) were carried out, whose increased values validated the importance of a slightly lipophilic character peculiar to the respective surfactants in the blend. %EE was first determined immediately after preparation, to assess the initial capacity of the niosomes to accommodate bioactive molecules and, in particular, the fungal dye. In the view of the four samples with niosomes containing bioactive molecules, the entrapment efficiencies (%EE) (Table 4) were identified as considerably high values, situating themselves within the 83.54–92.71% range. The findings specific to this study (Figure 2 and Table 4) evidenced an increase over time in niosomes size concomitant with the amount of encapsulated substance.

The highest entrapment efficiency value (Table 4), for the formulation obtained with the assistance of probe sonication, of 92.71% ± 0.01 (HLB = 8.42), was higher compared to the result of 91.5% ± 1.93 achieved by Bayındır & Yuksel [72] with paclitaxel (Span 20-HLB = 8.6). Beyond this, it was even proven to surpass the maximum value of 91.30% ± 0.008 obtained in the case of nimesulide by [93], using the same surfactant, both prior to and following the optimisation. This specific detail validates the fact that an HLB value corresponding to a domain that is only slightly lipophilic, around 8.6, generally drives the formation of niosomes with a maximal entrapment efficiency for the active substance.

Additionally, it may be observed that, although inferior to the entrapment efficiency corresponding to NA1-type niosomes, the value for NA3-type niosomes is greater than that of the niosomes made using bath sonication. This means, among other things, that the main reason the fungal pigment was immobilised was the use of the hand-shaking technique.

**Figure 11:**
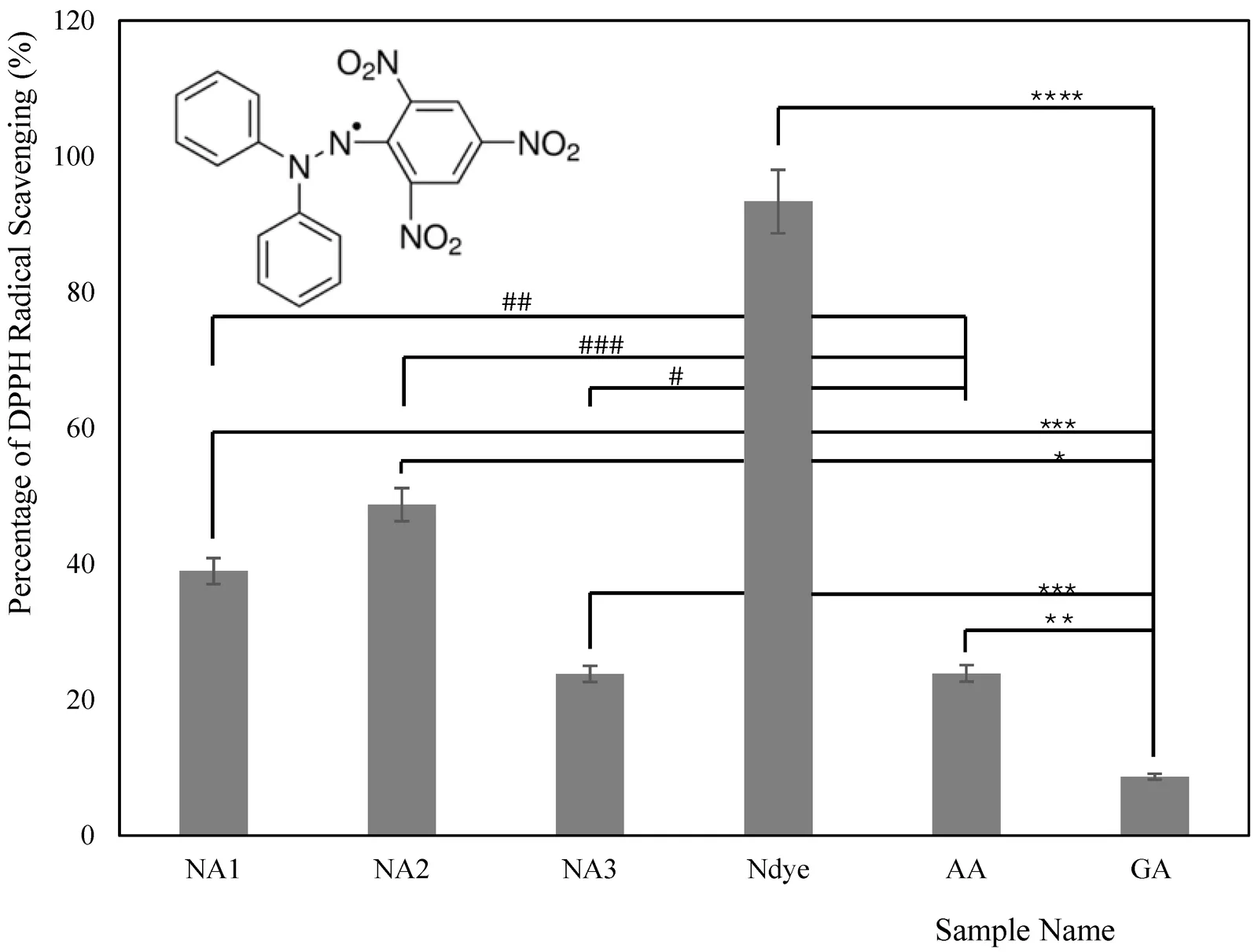
The antioxidant capacity profile of the blank and the three vitamin C niosomal formulations, following a 30-day period using the DPPH method, in comparison with that of 0.01% gallic acid (after a 20 min incubation). Data are the average of three independent experiments (mean ± SD; *n* = 3; * (*p* < 0.05), ** (*p* < 0.01), *** (*p* < 0.001), **** (*p* < 0.0001) indicate a significant difference from the control, and # (*p* < 0.05), ## (*p* < 0.01) and ### (*p* < 0.001) indicates significant difference from group NA. Also, the chemical structure of the DPPH stable free radical used to assess antioxidant activity is presented.

Regarding the antioxidant capacity assessment performed via the DPPH assay 30 days post-formulation (as shown in Figure 11), the analysis of the investigated samples revealed that their free radical scavenging capacity is primarily and directly influenced by the amount of free bioactive substance correlated with the specific preparation method employed.

The DPPH assay was selected as our primary screening tool because it is widely acknowledged in the literature as a robust, highly reproducible, and accurate method for evaluating the radical scavenging capacity of both hydrophilic and lipophilic antioxidants, making it highly suitable for our specific encapsulated formulations [94,95].

In such a context, the niosome formulation obtained through bath sonication (NA2 sample) showcased the greatest value in terms of the percentage of scavenged radical species, in comparison to GA, which is frequently used as a control antioxidant biomolecule [96]. Given that the DPPH alcohol solution was prepared with ethanol, these results highlight the effectiveness of the sonication formulation technique used in this study. Similarly, in the case of the AA niosomes prepared through the two isolated sonication types, values appeared that were relatively close to that of free vitamin C as reported by Bala et al. (2011) [97].

On the other hand, when discussing the niosomes prepared with the ultrasonication method involving the sonication bath (NA2), the results achieved in this research (Figure 8) are comparable to those reported in recent studies on niosomes obtained with TFH by Habibi et al. [98]. Our results are directly correlated with the lower entrapment efficiency of these niosomes; the higher amount of unencapsulated (free) ascorbic acid in the system reacted much faster with free radicals compared to the protected fraction in NA1, for example. Thus, these results show that the superior DPPH scavenging activity of NA2 reflects the higher fraction of free ascorbic acid resulting from its lower entrapment efficiency, rather than a technological advantage of the formulation technique. These aspects are also supported by the low Pearson linear correlation coefficients (Table 5). Likewise, the findings obtained in the course of this study reveal that the percentages of DPPH Radical Scavenging (%) for niosomes containing AA in highly similar concentrations are consistent with the results reported by Tavano et al. [99], who obtained multilamellar niosomes (MLVs) by the method involving the hydration of a lipid film.

Furthermore, the data presented in Figure 11 show that formulations NA1 and NA2 exhibit higher antioxidant activity than free ascorbic acid. This aspect can be mainly attributed to a synergistic effect between ascorbic acid and the non-ionic surfactants, which may possess a slight intrinsic antioxidant activity or structurally stabilise the active substance. The obtained values demonstrated that ascorbic acid remains active under the processing conditions during encapsulation into niosomes. This strengthens the conclusion regarding the preservation of the active substance’s bioactive function post-processing, as well as the enhancement of its stability and antioxidant properties through encapsulation. Additionally, formulation NA3 shows a practically identical activity (within statistical error limits) to that of free ascorbic acid. This behaviour suggests a very slow release of the active substance from the niosomal matrix or an increased membrane rigidity, which hinders the direct interaction of the encapsulated ascorbic acid with the DPPH radical during the assay [83,100,101].

On the other hand, in the case of the Ndye formulation, a considerably higher antioxidant activity is observed compared to both ascorbic acid and gallic acid. This aspect can likely be attributed primarily to a synergistic effect between the compounds with antioxidant properties in the fungal dye composition and the non-ionic surfactants.

For a comparative and overall analysis of the niosome formulations, a Pearson correlation matrix was developed (Table 5). Pearson’s coefficients (*r*) were used to evaluate the relationships between the properties of the analysed vesicle systems. The correlation coefficients calculated for the parameters monitored over a 30-day period revealed significant correlations. Specifically, a strong positive correlation was observed regarding the variation over time of key vesicular properties, such as particle size. Conversely, a strong negative correlation was noted for pH. Additionally, strong negative and positive correlations were found for entrapment efficiency and antioxidant activity, respectively.

In this context, the data presented in Table 5 show that the use of the Pearson correlation matrix represents a valuable statistical tool in the study of niosomes. These results provide a rigorous perspective on optimising the encapsulation of bioactive molecules.

## 3. Materials and Methods

### 3.1. Materials

Regarding the development of niosomes-blank, containing ascorbic acid (UCB S. A., Brussels, Belgium) (100 mg/mL) and containing yellow fungal dye (abundant in carotenoids) extracted from an *Epicoccum nigrum* strain and previously studied with respect to its antibacterial aspect and antioxidant characteristics [81], a previously investiged surfactant mixture [10] formed from sorbitan monooleate, Span 80 and polyoxyethylene (20) sorbitan monolaurate, Tween 20 (Sigma-Aldrich, St. Louis, MO, USA) (HLBmixture = 8.42) with cholesterol (Fluka Chemicals, Buchs, Switzerland) was utilised, in a molar ratio of 2:1:1 aligning with the indications of Khan et al. [51] with certain modifications, as detailed in Table 6. 

Aside from these, glyceryl stearate, SE (Self-emulsifying, Emulgator Bella) (Ellemental, Oradea, Romania), was used as a secondary excipient targeting stabilisation and alteration of membrane rigidity. For the aqueous phase a Gomori phosphate-buffered solution with a pH of 7.4 was utilised.

### 3.2. Niosomes Preparation Methods

In aiming to prepare niosomes containing vitamin C or the fungal dye individually, three methods were involved: probe sonication and bath sonication, respectively, and thin-film hydration (TFH), in the manner they are presented in Table 7.

The NB and NA1 niosomes formulas were obtained through the application of the probe ultrasonication method using a Bandelin Sonopuls HD 3100 sonicator. Equivalent to the procedure pursued by Khan et al. [51], a pulsed mode was employed with 5 s of sonication and 1 s of pause, for a duration of three minutes, at an amplitude of 30% and a temperature of 22 °C; the three-minute sonication interval was then supplemented by 1 min (35 kHz) in order to achieve an advanced degree of homogeneity. In the case of bath sonication, used for generating the NA2 sample, the ultrasonic bath (Elma Transonic T310, 35 kHz), filled with distilled water and ice so that the structural integrity of the active substance from the physical mixture could be preserved, was set at 10 min to optimise the entrapment efficiency [52].

The TFH protocol used for the preparation of NA3 and Ndye was adapted from the method established by Laslău et al. [55]. For this purpose, with a focus on achieving the Ndye formulation, the non-ionic surfactants and cholesterol were dissolved in 5 mL of pure ethanol, with the fungal colourant subsequently added to the ethanolic phase, after which the solvent was evaporated using a DLab RE-100 Pro rotary evaporator equipped with a DLab VC100 vacuum controller. Evaporation was carried out at parameters set at 200 rpm and 60 °C at a pressure of 156 mb (millibars), for a 10 min time span, resulting in the formation of a lipidic thin film which was coloured in the case of the Ndye formula. For the assessment of the formed film’s quality, we used a working technique that was previously reported by Thabet et al. [102]. For film hydration, a volume of 25 mL phosphate-buffered solution having a pH of 7.4 was added to it. The homogenisation was then carried out in a similar manner to the working protocol used in the production of the NA3 sample, with the exception that the procedure was continued for a 30 min interval, divided into two rounds of 15 min each, so that the fungal dye sample would have the necessary time for a homogeneous dispersion to form.

### 3.3. Niosome Physicochemical and Morphological Analysis

With regard to the preliminary physico-chemical characterisation of the niosomes, these were first viewed under an optical microscope, and subsequently their size was determined, along with their distribution. Because pH is significant in the characterisation of niosomes and products with cosmeceutical importance, with a focus on determining its values in the case of niosomal dispersions, the Consort (Ghent, Belgium) C862 pH metre equipped with a glass electrode [103] was used. The analyses were performed in triplicate and the average pH ± SD was reported.

As for the investigationof the niosomes with regard to morphological characterisation and the determinationof physical parameters, we used a Euromex Novex B (Netherlands) optical microscope with 4× and 10× objectives, with the eyepiece using 10× magnification, with the micrographs captured with the aid of a smartphone with the built-in AI Camera option activated for image quality enhancement; the use of external artificial intelligence (AI)-based platforms for editing them was excluded altogether. The determination of niosomes dimensions and vesicular size distribution was carried out individually by analysing the micrographs taken, using NIH ImageJ software (Version 1.54g; National Institutes of Health, Bethesda, MD, USA) based upon micrographs obtained on aqueous diluted fractions (1:10 by volume) in conformity with the procedure used by Ababei et al. [104].

The dimensions of the vesicles were determined by taking into consideration the field of view (FOV) diameter for the 10×/40× (×10 and ×40 magnification) objectives and the 10×/18 eyepiece in accordance with the product data sheet [105] and user’s manual [106]. The FOV diameter was calculated using relation (1) in accordance with the indications of Spring, Kenneth et al. [107].

Field of view (FOV) diameter (mm) = (Field number)/(Objective magnification)(1)

With respect to infrared spectroscopy, the FT-IR Thermo Scientific Nicolet iS-50 spectrometer set to attenuated total reflectance ATR module, equipped with OMNIC software, and featuring a diamond disc as part of its equipment, was used.

The niosomal formulations were stored under ordinary temperature conditions in order to observe their stability over time, in comparison with liposomal vesicles, which are known to be less stable at such temperatures [108].

### 3.4. Determination of Niosome Entrapment Efficiency (EE%)

In order to determine the entrapment efficiency (EE%) of the niosomes, we used the protocol elaborated by Vahidi et al. [109], with some modifications. Briefly, 1 mL of niosomal suspension was diluted with 1 mL phosphate-buffered solution, or alternatively with ethanol in the case of Ndye formulation (1:1 ratio), after which it was centrifuged using an LBX Instruments MC-7000 minicentrifuge at 7000 rpm for ten minutes. The completeness of the separation was demonstrated by a perfectly clear supernatant post-centrifugation, characterised by a total absence of turbidity, confirming that all the micron-sized vesicles were successfully sedimented. After a prior dilution by an excess of 2 mL solvent (to achieve a niosomal suspension: solvent ratio (*v*/*v*) = 1:3), the appropriate absorbance values were assessed using a RayLeigh UV-1801 UV-Vis spectrophotometer (China), separately for ascorbic acid at λ = 263 nm [55], whereas a λ = 429 nm was set in the case of fungal colourant [81]. This mild centrifugation protocol was strictly maintained to prevent mechanical disruption and subsequent dye leakage, which often occurs when large vesicle structures are subjected to high-speed ultracentrifugation.

Following the prior establishment of the calibration curves for the individual compounds, the entrapment efficiency was determined according to relation (3) proposed by Vahidi et al. [109]:

EE % = (w_encapsulated_/w_initial_) × 100 (2)

where w_encapsulated_ is the weight of the encapsulated substance, whereas w_initial_ is the weight of the substance introduced in the sample. The measurements were carried out in triplicate.

### 3.5. Determination of Antioxidant Activity by DPPH Assay

The DPPH assay was selected due to its operational simplicity, high stability of the radical, and widespread validation in assessing entrapment efficiency of bioactive molecules [95,99,110].

For the determination of the antioxidant capacity using the DPPH assay, a 3.4 mg/100 mL (0.086 M) methanolic solution of 2,2-diphenyl-1-picrylhydrazyl (DPPH) was used, with the experimental determinations having been carried out in accordance with the method utilised by [96,111], with minor modifications. For the sample to be analysed, 1 mL of niosomal formulation plus 2 mL of DPPH were mixed, with the control being treated similarly, albeit using 2 mL of absolute methanol instead of DPPH. In the case of the blank, the working procedure was carried out analogously to the control sample, although by combining 1 mL of DPPH with 2 mL of methanol. For the chemical reaction to take place, the samples were kept for a duration of 20 min under dark conditions, with the separate absorbances thereafter read at a maximum wavelength of λ_max_ = 517 nm, with the precise aim of determining the antioxidant capacity, in the way described in the following calculation formula:

DPPH radical scavenging capacity (%) = [1 − (A_sample_ − A_control_)/A_blank_] × 100 (3)

where A_sample_ represents the absorbance of the sample–DPPH solution mixture, A_control_ represents the sample–MeOH couple and A_blank_ the blank sample absorbance.

### 3.6. Statistical Analysis

All data were expressed as the mean ± standard deviation (SD). All the experiments were performed with *n* = 3 independent replicates. The statistical analysis was carried out using Statistical Product and Service Solutions software (SPSS for Windows, Version 10.0; SPSS Inc., Chicago, IL, USA). The differences between the samples were analysed using one-way analysis of variance (ANOVA) followed by Tukey’s HSD Test. The experiments were analysed by Pearson’s correlation coefficient. A difference was considered statistically significant if *p* < 0.05.

## 4. Conclusions

The present investigation outlines a clear dependence of the stability and antioxidant capacity of the active molecules within the niosomal composition on both the surfactant ratio investigated in this study and the specific preparation techniques employed: namely, two distinct ultrasonication techniques combined with the thin-film hydration (TFH) procedure. The utilisation of a Span 80 and Tween 20 non-ionic surfactant mixture of distinct types presenting an HLB value close to 8.4 confirms the increased entrapment efficiency and the structural rigidity required for their stability. In addition, based on the use of a Span 80-Tween 20 blend in a 2:1 molar ratio, similar to the weight ratio of 80:20 with a ratio of Span 80 and Tween 60 [35], the possibility of their functionality as spanlastics might be brought into discussion.

The increase in niosomes dimensions over time, following the encapsulation of a certain number of bioactive molecules within their structure, could be cumulatively attributed to the formulation techniques, molecular interactions, structural organisation changes in the bilayer and the hydration phenomena. In contrast, among all the niosomal formulations, it was the one obtained using bath sonication that proved to be the most stable after the blank one. This trend was observed because the niosomes prepared via bath sonication exhibited the strongest antioxidant activity, despite displaying the lowest biomolecule entrapment efficiency. In addition, the niosomal systems formed via the thin-film hydration method, despite exhibiting minimal antioxidant activity, offer the highest encapsulation yield compared to those generated through bath sonication. Lastly, it can be concluded that the synergy between the two procedures guarantees the formation of a pair of niosomal systems showcasing optimal stability and increased biological performance in concert.

Our findings reveal the antioxidant activity of certain bioactive substances, ascorbic acid being one example. Therefore, the DPPH radical scavenging activity could increase considerably depending on the formulation conditions.

Ultimately, under the present experimental conditions, the bath sonication method may be utilised for the initial preparation of niosomes, despite its lower entrapment efficiency. Concurrently, bath sonication may subsequently be applied for the reduction in vesicle size and the achievement of a uniform distribution, concomitant with the enhancement of free radical scavenging capacity, attributed to the stabilisation of the bioactive compound.

Besides that, fungal pigments can be successfully utilised in the formulation of various niosomes. Aside from their colouring properties, these biocolorants enhance the antioxidant capacity of such colloidal systems due to their high carotenoid content.

As such, in our future studies we foresee expanding the research in this direction. Additionally, in the future, we propose to synergistically encapsulate hydrophilic and lipophilic substances. In parallel, future efforts aim to encapsulate a broader range of antioxidant molecules into advanced vesicular systems, including bilosomes, transfersomes, liponiosomes, and ethoniosomes. Furthermore, the development of cosmeceutical products, such as natural extract-based creams and gels, is envisioned, as the feasibility of encapsulation within these systems [86] is already evidenced by the relatively large sizes of the obtained vesicles.

## Figures and Tables

**Table 1:** Schematisation for a range of methods used in the preparation of niosomes containing encapsulated substances.

Curr. No.	Name of the Method	Advantages	Disadvantages	Entrapped Substance	Reference
1.	Thin-film hydration	Recoverable solvents	Toxic organic solvent usage	5-Fluorouracil Crocin Minoxidil	[53,54,56]
2.	Reverse-phase evaporation	Removable solvents	Toxic solvent involvement	Acetazolamide	[44,57]
3.	Transmembrane pH gradient	Uplifted vesicle stability maintainable in conditions of elevated temperatures	Organic solvents utilisation	Mupirocin	[42,45,47]
4.1.	Probe sonication	Green procedure Particles with curved diameter length	High energy consumption	Diacerein Rifampicin Ceftriaxone	[53,54]
4.2.	Bath sonication	Raised entrapment efficiency Lower particle size Niosomal formulation optimisation	Decreases in entrapment efficiencies at greater ultrasonication durations	Levamizole Pilocarpine HCl ^1^	[55,58]

^1^ The specific formulations were initially obtained through ether injection in the study conducted by Owodeha-Ashaka et al. [58], and were only later optimised with the assistance of probe sonication.

**Table 2:** The average pH variation in time indicated by niosomes produced through use of various methods. The results represent mean ± SD of triplicate readings.

Sample/pH	Initial	Final	*p*-Value
NB	6.947 ± 0.003	6.837 ± 0.022	<0.05
NA1	6.868 ± 0.008	6.779 ± 0.015	<0.01
NA2	6.868 ± 0.045	6.837 ± 0.010	<0.01
NA3	1.966 ± 0.094	1.902 ± 0.008	<0.001
Ndye	2.017 ± 0.019	1.956 ± 0.119	<0.05

Statistically significant difference (*p* < 0.05).

**Table 3:** Identification of functional groups and their corresponding FTIR wavenumbers (cm^−1^) across samples.

Peak Wavenumbers (cm^−1^) and Sample Occurrences	Functional Group	Reference
3319.63 (NB), 3319.63 (NA_1_), 3316.42 (NA_2_), 3331.22 (NA_3_), 3331.58 (Ndye), 3420.48 (Chol)	-OH Hydrogen bond stretch	[38]
3523.88, 3406.09, 3309.43, 3206.75 (AA)	-OH alcohol stretch	[88]
2930.48 (Chol)	-CH_2_- asymmetric stretch	[89]
2900.14 (Chol)	>CH- asymmetric stretch	[88]
2866.08 (Chol)	-CH_3_ symmetric stretch	[89]
2847.72 (Chol)	-CH_2_ symmetric stretch	[88]
1752 (AA)	C=O stretch (Lactone ring-penta-atomic ring)	[91]
1652.02 (AA), 1636.09 (NB), 1636.09 (NA_1_), 1636.08 (NA_2_), 1636.03 (NA_3_), 1636.01 (Ndye)	Olefinic C=C stretch	[91]
1464.72 (Chol)	-CH_2_- bending	[90]
421.05 (NA_3_)	Alkenic *cis*-C-H out-of-plane bend	[88]

**Table 4:** The %EE values peculiar to the four bioactive compound niosomal formulations. The results represent mean ± SD of triplicate readings.

Sample Name	%EE Value
NA1	92.71 ± 0.01 ^a^
NA2	72.83 ± 0.02 ^b^
NA3	86.38 ± 0.05 ^c^
Ndye	83.54 ± 0.08 ^b^

The mean values with different small letters within a column indicate significant differences: *p* < 0.05 (a), *p* < 0.01 (b), *p* < 0.001 (c).

**Table 5:** Pearson correlation coefficients (*r*) and *p*-values for niosomal formulations ^a,b,c,d^.

	t *	pH	d **	% EE	DPPH
**^a^** **NA1**					
**t**	1 (…)				
**pH**	**−0.922** (*0.0053*)	1 (…)			
**d**	**0.998** (*0.0042*)	−0.943 (*0.259*)	1 (…)		
**EE%**	**0.992** (*0.0041*)	**−0.963** (*0.034*)	**0.998** (*0.012*)	1 (…)	
**DPPH**	−0.992 (*0.603*)	−0.963 (*0.182*)	0.997 (*0.348*)	**0.998** (*0.048*)	1 (…)
**^b^** **NA2**					
**t**	1 (…)				
**pH**	**−0.894** (*0.027*)	1 (…)			
**d**	**0.896** (*0.0006*)	**−0.998** (*<0.0001*)	1 (…)		
**EE%**	−0.847 (*0.204*)	0.995 (*0.105*)	−0.994 (*0.059*)	1 (…)	
**DPPH**	**−0.997** (*0.0033*)	0.873 (0.323)	−0.877 (*0.259*)	**0.824** (*0.021*)	1 (…)
**^c^** **NA3**					
**t**	1 (…)				
**pH**	**−0.829** (*0.0062*)	1 (…)			
**d**	**0.941** (*0.0056*)	−0.969 (*0.158*)	1 (…)		
**EE%**	−0.996 (*0.125*)	0.853 (*0.501*)	−0.955 (*0.098*)	1 (…)	
**DPPH**	**−0.926** (*<0.0001*)	0.979 (0.375)	**−0.998** (*<0.0001*)	**0.942** (*0.0073*)	1 (…)
**^d^** **Ndye**					
**t**	1 (…)				
**pH**	**−0.996** (*0.0014*)	1 (…)			
**d**	0.805 (*0.0166*)	−0.849 (*0.213*)	1 (…)		
**EE%**	−0.896 (*0.016*)	0.927 (*0.446*)	**−0.985** (*0.034*)	1 (…)	
**DPPH**	**−0.952** (*0.0028*)	0.973 (*0.145*)	−0.948 (*0.052*)	**0.989** (0.031)	1 (…)

* t—time, days; ** d—diameter, μm; values represent Pearson’s correlation coefficients (*r*) with *p*-values in parentheses (where *p* is italicised as statistical variables). Coefficients with statistical significance (*p* < 0.05) are indicated in bold; *n* = 3.

**Table 6:** Formulation of ascorbic acid and fungal dye niosomes.

Niosomes Acronym *	Ingredients Amount						
	Span 80 (mg)	Tween 20 (mg)	Chol (mg)	Bella Emulsifier (mg)	Phosphate Buffer (mL)	AA (mg)	Fungal Dye (mg)
NB	171.4	245.6	77.3	12.98	25	-	-
NA_1_						2.5	-
NA_2_						2.5	-
NA_3_						2.5	-
Ndye						-	2.5

* NB-Blank niosomes (without bioactive molecules); NA_i_—Niosomes containing vitamin C (0.1 mg/mL), i = 1–3 depending on the method involved in their preparation (meaning to be found in Table 7); Ndye—Niosomes containing fungal dye.

**Table 7:** Preparation methods respective of niosomal formulations.

Niosomes Acronym	Involved Method
NB *	Probe sonication
NA1 **	Probe sonication
NA2 **	Bath sonication
NA3 **	Thin-film hydration (TFH)
Ndye *	Thin-film hydration (TFH)

* identical meaning as in Table 6. ** NA—Niosomes with AA (0.1 mg/mL).

## Data Availability

The data presented in this study are available upon request.

## Supplementary Materials

The following supporting information can be downloaded at: https://www.mdpi.com/article/10.3390/ph19081241/s1, Table S1: Over-view with a selection of types of transport vesicles featuring a lipid bilayer, connected to niosomes; Table S2: Schematisation of a range of methods used in the preparation of niosomal vesicles containing encapsulated sub-stances.
