# Supplementary material for: Bioactive Molecules Niosomes Containing a Span 80 and Tween 20 Surfactant Mixture: Obtaining, Physico-Chemical Characterisation and Stability
Source: Pharmaceuticals (Basel). 2026 Aug 6;19(8):1241. doi: 10.3390/ph19081241 (PMC13516028; doi:10.3390/ph19081241)
Supplement: Supplementary file 1 [file pharmaceuticals-19-01241-s001.zip › pharmaceuticals-4369244-supplementary.pdf]

Table S1. Overview with a selection of types of transport vesicles featuring a lipid bilayer, connected to niosomes.

Transport vesicle type	Benefits	Limitations	References
Liposomes	Increased stability Facile delivery of hydrophobic substances	Constrains regarding the increase in polydispersity, strictly dependent on the presence of non-ionic surfactant for a controlled dimensional profile. Restricted applicability	[1,2]
Transfersomes	Deformability during delivery	Hydrophilic substance leakage	[3,4]
Ethosomes	Enhanced biodegradability	Irritability caused by ethanol, particularly toward to weeping eczemas	[5,6]
Transethosomes	Improved fluidity compared tothat of ethosomes	Skin irritability, similar to that of ethosomes	[7–9]
Chitosomes	Strengthened stability Formation of a protective layer for the gastrointestinal tract	Curtailed entrapment efficiency due to the impact of the acidic environment	[10,11]
Bilosomes	Structural integrity inside the gastrointestinal (GI) milieu Attenuated gastric mucosa irritability	Weak immobilisation of active anionic agents Cytotoxicity manifested by bile salts	[12–14]
Ethoniosomes	Amplified membrane elasticity	Instability at elevated temperatures	[15,16]
Liponiosomes	Improved therapeutical efficacy and stability compared with liposomes and niosomes	Reduced commercial applications	[17,18]
Transniosomes	Increased deformability when crossing physiological barriers Considerable augmentation in antioxidant activity	Increased Polydispersity Index (PDI)	[19–21]

Structurally, liposomes incorporate lecithins (phosphatides)—such as soy phosphatidylcholine—and cholesterol within the membrane bilayer to ensure stability and rigidity. Their enhanced counterparts integrate non-ionic surfactants (e.g., Span, Tween, and Brij series) to simultaneously increase both the Zeta potential and entrapment efficiency.These, used in quantities of 15 ÷ 20%, drive the formation of elastic liposomes, transfersomes that are deformable throughout delivery [1,3]. Alternatively, edge-activating molecules, such as ionic surfactants stearylamine and sodium lauryl sulfate, bile salts, such as sodium cholate or deoxycholate, may be used. By replacing the phospholipids contained in transfersomes with non-ionic tensioactives such as those of the Span series, niosomal spanlastics are developed, playing an adjunct role in the ocular delivery of ketoconazole, extending the importance of non-ionic surfactants blending in this case [22].

With regard to biodegradable vesicles, the ethosomes [5] and their elastic variant of transethosomes [7] represent liposomes containing ethanol in their structure at ratios of 30 – 40 % making them readily processable by the cell systems from the human body. Not only could the two varieties be utilised in transdermal delivery in the case of anti-gout cataplasms [6], but also in dermal

delivery in the case of the anti-fungal conazoles excluding the possibility of deterioration during this process [9]; the niosomal version named ethoniosomes, facilitates pulmonary anticancer action with lipophilic stilbenes [15] along with diminishing the intraocular pressure in the case of prednisolone [16]. With the aid of edge-activating agents from ethoniosomes, the transethoniosomes complement the fluidity of their membranes with the quality of elasticity, strengthening the anti-inflammatory effect of inclusion complexes formed by inserting gingerols into β-cyclodextrins (β-CD) [23].

Among gastrointenstinally non-irritating vesicles, bilosomes and chitosomes, are recognised, which may be either liposomes [11,24] or niosomes [10,13]. Both include bile salts in their bilayer, or alternatively are coated with a protective layer formed by either chitosan or a derivative of chitosan, namely tripolyphosphate (TPP). In this way, there is a considerable increase in bioavailability and stability of the conventional vesicles within the gastrointenstinal (GI) tract [12,14].

Through the combination of the structural constituents mutually present in liposomes and niosomes, liponiosomes enhance the already existing benefits shown in the two typical types of lipid bilayer vesicles, with the ratios between lecithins and non-ionic surfactants being inherently reversed compared to that of transfersomes[19,25]. Besides, through encapsulation, the inhibitory capacity against *Aspergillus spp*. shown by antifungal plant extracts, a fact observed by Mohammadi et al. ~~(2019)~~ [25], and, likewise, the anticancer efficacy presented by bioactive molecule conjugates with zinc (Zn) metal-organic frameworks (ZMOF)[18] are both potentiated. In this case, the integration of edge activators from transfersomes into liponiosomes, even in proportions of only 5%, produces the formation of transniosomes with a lipid bilayer deformability superior to that of any of the transfersomes, liposomes, niosomes, and implicitly transethosomes during transport, evincing their usefulness against glaucoma [19], diabetes mellitus [20] and ultimately skin anti-ageing [21].

Table S2. Schematisation of a range of methods used in the preparation of niosomal vesicles containing encapsulated substances.

Curr. No.	Name of the method	Advantages	Disadvantages	Entrapped substance	Reference
1	Nitrogen N_2 gas bubbling	It does not require the use of organic solvents.	Limited practical expertise	Proteins [1]	[26–28]
2.1	Lipid injection (quick)	The use of organic solvents is not necessary. Rapid procedure	Vulnerability in terms of thermolabile drugs	Tetanic (TT) and Difteric (DT) Toxoids	[26,29,30]
2.2	Ether injection (slow)	Lipid-phase pre-heating is not required.	Inadvisable for thermolabile drugs Organic solvent residues are difficult to remove	Mupirocin Curcumin	[26,31,32]
3.	Ball milling	Diminished particle sizes Minimised polydispersity Environmentally friendly method	Necessity of a specialised device	Melatonin	[28,33]

[1] The formed niosomes and liposomes in the study conducted by Talsma et al. (1994) have in either cases been of blank type, not to mention the encapsulation of said biomolecules solely existing to a prospective extent nowadays.

The nitrogen bubbling method, used for simultaneously producing liposomes and niosomes, does not necessitate solvents,nor even detergents and is considered as an environment-friendly means of generating them [26, 27]. There is a higher possibility of using such a method, thereby precluding a

great number of risks pertaining the ultimate gas (N_2) entrapment. Thiscand be atributed, on the one hand, to the possibility of intermediary nitrogen encapsulation into micelles followed by its expulsion, preceded by their transformation into bilayers that are ultimately curved into the desired vesicles. On the other hand, the use of this methods results in the creation of a range of stable vesicular systems in which the denaturation of certain substances, mostly proteins, is prevented throughout transport. In accordance to the studies carried out by Mawazi & Widodo et al. [28] it can be finally inferred from the fact that the delivery of volatile bioactive substances can be developed in a wide range of directions, that the presence of nitrogen exclusively facilitates vesicle formation.

In particular case of the rapid lipid injection method, the preheated surfactants: cholesterol mixture is introduced into the aqueous phase containing biologically active molecules dispersed in a buffer solution, without prior lipid dissolution beingnecessary, so that both phases can be blended [30]. In the slower version of this procedure, in which the lipids are contained in the ether phase, the ether injection contributes to the formation of LUV-type niosomes, similarly to the rapid variant, albeit with the additional need for rotary evaporator involvement. [26].

The state-of-the-art research performed by Temprom et al. [33] on the preparation of SUV-type niosomes, investigated techniques such as ball milling. In this technique used for the making of niosomal vesicles,aseptic ZrO_2 balls are employed for grinding the bioactive compounds with their degree of uniformity being increased due to the higher milling speed and, especially, the smaller ball sizes [28].

Supplementary references

1. Tsichlis, I.; Manou, A.-P.; Manolopoulou, V.; Matskou, K.; Chountoulesi, M.; Pletsa, V.; Xenakis, A.; Demetzos, C. Development of Liposomal and Liquid Crystalline Lipidic Nanoparticles with Non-Ionic Surfactants for Quercetin Incorporation. *Materials* **2023**, *16*, 5509, doi:10.3390/ma16165509.
2. Shaikh Hamid, M.S.; Bakal, R.L.; Kohale, N.B. A Comprehensive Review on Liposomes: As a Novel Drug Delivery System. *GSC Biol. Pharm. Sci.* **2024**, *27*, 199–210, doi:10.30574/gscbps.2024.27.1.0121.
3. Matharoo, N.; Mohd, H.; Michniak-Kohn, B. Transferosomes as a Transdermal Drug Delivery System: Dermal Kinetics and Recent Developments. *WIREs Nanomed Nanobiotechnol* **2024**, *16*, e1918, doi:10.1002/wnan.1918.
4. Webster, T.; Singh, S.; Vardhan, H.; Kotla, N.; Maddiboyina, B.; Sharma, D. The Role of Surfactants in the Formulation of Elastic Liposomal Gels Containing a Synthetic Opioid Analgesic. *IJN* **2016**, 1475, doi:10.2147/IJN.S100253.
5. Pandey, V.; Golhani, D.; Shukla, R. Ethosomes: Versatile Vesicular Carriers for Efficient Transdermal Delivery of Therapeutic Agents. *Drug Delivery* **2015**, *22*, 988–1002, doi:10.3109/10717544.2014.889777.
6. Wei, Y.-Y.; Zhang, Y.; Lu, X.; Zhou, J.; Cheng, H.-W.; Liu, Y.-Y.; Zhang, H.; Chen, W. Novel Colchicine Ethosomes Cataplasm for the Treatment of Acute Gouty Arthritis. *Colloids and Surfaces B: Biointerfaces* **2025**, *254*, 114776, doi:10.1016/j.colsurfb.2025.114776.
7. Seenivasan, R.; Halagali, P.; Nayak, D.; Tippavajhala, V.K. Transethosomes: A Comprehensive Review of Ultra-Deformable Vesicular Systems for Enhanced Transdermal Drug Delivery. *AAPS PharmSciTech* **2025**, *26*, 41, doi:10.1208/s12249-024-03035-x.
8. Zhan, B.; Wang, J.; Li, H.; Xiao, K.; Fang, X.; Shi, Y.; Jia, Y. Ethosomes: A Promising Drug Delivery Platform for Transdermal Application. *Chemistry* **2024**, *6*, 993–1019, doi:10.3390/chemistry6050058.
9. Song, C.K.; Balakrishnan, P.; Shim, C.-K.; Chung, S.-J.; Chong, S.; Kim, D.-D. A Novel Vesicular Carrier, Transethosome, for Enhanced Skin Delivery of Voriconazole: Characterization and in

Vitro/in Vivo Evaluation. *Colloids and Surfaces B: Biointerfaces* **2012**, *92*, 299–304, doi:10.1016/j.colsurfb.2011.12.004.
10. Meoli, C.M.; Riccardi, D.; Baldino, L. Optimization of Chitosan-Coated Niosomes Encapsulating Doxycycline Hyclate for pH-Responsive Drug Release. *Ind. Eng. Chem. Res.* **2025**, *64*, 6292–6301, doi:10.1021/acs.iecr.4c04456.
11. Esposto, B.S.; Pinho, S.G.B.; Thomazini, M.; Ramos, A.P.; Tapia-Blácido, D.R.; Martelli-Tosi, M. TPP-Chitosomes as Potential Encapsulation System to Protect Carotenoid-Rich Extract Obtained from Carrot by-Product: A Comparison with Liposomes and Chitosomes. *Food Chemistry* **2022**, *397*, 133857, doi:10.1016/j.foodchem.2022.133857.
12. Ahmad, J.; Singhal, M.; Amin, S.; Rizwanullah, Md.; Akhter, S.; Kamal, M.A.; Haider, N.; Midoux, P.; Pichon, C. Bile Salt Stabilized Vesicles (Bilosomes): A Novel Nano-Pharmaceutical Design for Oral Delivery of Proteins and Peptides. *CPD* **2017**, *23*, 1575–1588, doi:10.2174/1381612823666170124111142.
13. Binsuwaidan, R.; Sultan, A.A.; Negm, W.A.; Attallah, N.G.M.; Alqahtani, M.J.; Hussein, I.A.; Shaldam, M.A.; El-Sherbeni, S.A.; Elekhnawy, E. Bilosomes as Nanoplatform for Oral Delivery and Modulated In Vivo Antimicrobial Activity of Lycopene. *Pharmaceuticals* **2022**, *15*, 1043, doi:10.3390/ph15091043.
14. Abdel-moneum, R.; Abdel-Rashid, R.S. Bile Salt Stabilized Nanovesicles as a Promising Drug Delivery Technology: A General Overview and Future Perspectives. *Journal of Drug Delivery Science and Technology* **2023**, *79*, 104057, doi:10.1016/j.jddst.2022.104057.
15. Hanafy, N.A.N.; Abdelbadea, R.H.; Abdelaziz, A.E.; Mazyed, E.A. Formulation and Optimization of Folate-Bovine Serum Albumin-Coated Ethoniosomes of Pterostilbene as a Targeted Drug Delivery System for Lung Cancer: In Vitro and in Vivo Demonstrations. *Cancer Nano* **2023**, *14*, 49, doi:10.1186/s12645-023-00197-4.
16. Gaafar, P.M.E.; Abdallah, O.Y.; Farid, R.M.; Abdelkader, H. Preparation, Characterization and Evaluation of Novel Elastic Nano-Sized Niosomes (Ethoniosomes) for Ocular Delivery of Prednisolone. *Journal of Liposome Research* **2014**, *24*, 204–215, doi:10.3109/08982104.2014.881850.
17. Tripathi, M.A. Liponiosomal Drug Delivery System – A Review. *JPSBR* **2013**, *3*, 100–104.
18. Alharbi, H.M.; Eldin, Z.E.; Taha, M.; Elbeltagi, S. Preparation, Characterization, and Anticancer Evaluation of Polydatin Conjugated with Zinc MOF and Encapsulated by Liponiosomes as a Potential Nanotool-Induce Apoptosis. *Journal of Molecular Structure* **2024**, *1315*, 138982, doi:10.1016/j.molstruc.2024.138982.
19. Srivastava, V.; Patil, R.K.; Mehra, N.K. A One-Platform Comparison Study of Brinzolamide-Loaded Liposomes, Niosomes, Transfersomes, and Transniosomes for Better Management of Glaucoma. *International Journal of Pharmaceutics* **2024**, *666*, 124741, doi:10.1016/j.ijpharm.2024.124741.
20. Abdelwahab, A.; Abdelmohymen, A.; Mostafa, N.; Magdy, G.; Mazyed, E. Formulation of Deformable Liponiosomal Hybrid of Repaglinide: In Vitro Characterization and Evaluation of the Anti-Diabetic Effect. *IJN* **2023**, *Volume 18*, 7417–7440, doi:10.2147/IJN.S434840.
21. Eaknai, W.; Bunwatcharaphansakun, P.; Phungbun, C.; Jantimaporn, A.; Chaisri, S.; Boonrungsiman, S.; Nimmannit, U.; Khongkow, M. Ethanolic Fenugreek Extract: Its Molecular Mechanisms against Skin Aging and the Enhanced Functions by Nanoencapsulation. *Pharmaceuticals* **2022**, *15*, 254, doi:10.3390/ph15020254.
22. Kakkar, S.; Kaur, I.P. Spanlastics—A Novel Nanovesicular Carrier System for Ocular Delivery. *International Journal of Pharmaceutics* **2011**, *413*, 202–210, doi:10.1016/j.ijpharm.2011.04.027.
23. Mazyed, E.A.; Badria, F.A.; ElNaggar, M.H.; El-Masry, S.M.; Helmy, S.A. Development of Cyclodextrin-Functionalized Transethoniosomes of 6-Gingerol: Statistical Optimization, In Vitro Characterization and Assessment of Cytotoxic and Anti-Inflammatory Effects. *Pharmaceutics* **2022**, *14*, 1170, doi:10.3390/pharmaceutics14061170.

24. Can, A.; Niaz, T.; Tyler, A.I.I.; Mackie, A.R. Impact of Bile Salts, Biopolymer Coatings, and Food Matrix on Bilosome-Mediated Delivery of Trans-Resveratrol. *Food Chemistry: X* **2026**, *34*, 103547, doi:10.1016/j.fochx.2026.103547.
25. Mohammadi, M.; Haghirosadat, B.F.; Larypoor, M.; Ehsani, R.; Yazdian, F.; Rashedi, H.; Jahanizadeh, S.; Rahmani, A. Synthesis, Characterization and Evaluation of Liponiosome Containing Ginger Extract as a New Strategy for Potent Antifungal Formulation. *J Clust Sci* **2020**, *31*, 971–981, doi:10.1007/s10876-019-01702-9.
26. Gao, S.; Sui, Z.; Jiang, Q.; Jiang, Y. Functional Evaluation of Niosomes Utilizing Surfactants in Nanomedicine Applications. *IJN* **2024**, *Volume 19*, 10283–10305, doi:10.2147/IJN.S480639.
27. Talsma, H.; van Steenbergen, M.J.; Borchert, J.C.H.; Crommelin, D.J.A. A Novel Technique for the One-Step Preparation of Liposomes and Nonionic Surfactant Vesicles without the Use of Organic Solvents. Liposome Formation in a Continuous Gas Stream: The "Bubble" Method. *Journal of Pharmaceutical Sciences* **1994**, *83*, 276–280.
28. Mawazi, S.M.; Ge, Y.; Widodo, R.T. Niosome Preparation Techniques and Structure—An Illustrated Review. *Pharmaceutics* **2025**, *17*, 67, doi:10.3390/pharmaceutics17010067.
29. Karim, K.; Mandal, A.S.; Biswas, N.; Guha, A.; Chatterjee, S.; Behera, M.; Kuotsu, K. Niosome: A Future of Targeted Drug Delivery Systems. *J Adv Pharm Technol Res* **2010**, *1*, 374, doi:10.4103/0110-5558.76435.
30. Gupta, R.K.; Varanelli, Carole L.; Griffin, Paul; Wallach, D.F.H.; Siber, G.R. Adjuvant Properties of Non-Phospholipid Liposomes (Novasomes®) in Experimental Animals for Human Vaccine Antigens. *Vaccine* **1996**, *14*, 219–225.
31. Akki, R.; Ramya, M.G.; Navyasri, K.; Kathirvel, S. Formulation and Evaluation of Mupirocin Nimosomal Gel forTopical Drug Delivery System. *IJPTR* **2020**, *13*, 7–17, doi:10.20902/IJPTR.2019.130202.
32. John, C.R.; Sailaja, A.K. A Curcumin Loaded Niosomes as Novel Drug Delivery System by Ether Injection Method. *Drug Discovery* **2023**, *17*, 1–6, doi:10.54905/disssi.v17i39.e19dd1918.
33. Temprom, L.; Krongsuk, S.; Thapphasaraphong, S.; Priperm, A.; Namuangruk, S. A Novel Preparation and Characterization of Melatonin Loaded Niosomes Based on Using a Ball Milling Method. *Materials Today Communications* **2022**, *31*, 103340, doi:10.1016/j.mtcomm.2022.103340.
